# Leveraging factors that control alveolar epithelial cell fate enables large-scale expansion for lung tissue engineering

**DOI:** 10.1172/JCI188701

**Published:** 2026-06-01

**Authors:** Lauren K. Rochelle, Rachael S. Van, Richard J. Ottman, Daren F. Robinson, Ashley R. Dockham, Amy K. Smith, Daniel P. Keeley, Jia C. Wang, Darell W. McCoy, Tyler R. Zimmerman, Bryan A. Fioret, Ryan W. Bonvillain, Thomas H. Petersen, Sarah S. Hogan, Laila C. Roudsari

**Affiliations:** 1 Regenerative Medicine Lab, United Therapeutics, Research Triangle Park, North Carolina, USA.; 2 Computational Sciences, United Therapeutics, Silver Spring, Maryland, USA.

**Keywords:** Cell biology, Pulmonology, Stem cells, Adult stem cells, Human stem cells, Organ transplantation

## Abstract

Alveolar type 2 cells (AT2s) are critical to lung regeneration, and the absence of large-scale methods to expand AT2s has hindered regenerative medicine efforts. We report a microcarrier-based, large-scale expansion method that was used to generate hundreds of billions of human AT2s. Through our process, expanded AT2s largely retained their phenotype. Furthermore, we showed that culture medium, substrate composition, and stiffness are all critical to the maintenance of AT2s. Finally, we showed that expanded AT2s can differentiate into alveolar type 1–like cells, both in vitro and in a decellularized porcine lung, demonstrating the utility of these cells for lung tissue engineering.

## Abstract 2

Uncovering critical factors that influence alveolar epithelial type 2 cell phenotype to overcome the longstanding AT2 expansion challenge and render lung tissue engineering feasible.

## Graphical abstract

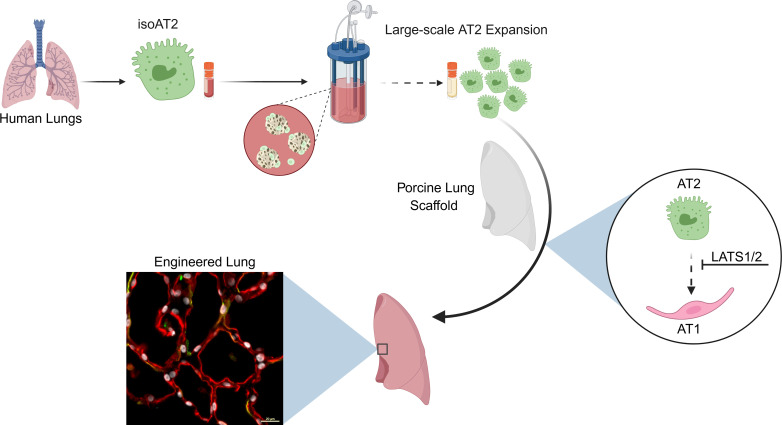

## Introduction

Alveolar type 2 cells (AT2s) are stem cells in the distal lung; extensive literature describes their capacity to proliferate and differentiate into alveolar type 1 cells (AT1s) in response to injury in vivo (1–5). Their progenitor competence, coupled with their capacity to secrete surfactant to keep alveoli patent, maintain an osmotic gradient to prevent edema, and modulate the immune system to support host defense, gives them great potential for regenerative medicine therapeutics (6–8). Implantation of AT2s has been shown to positively effect outcomes in animal models of acute lung injury and lung fibrosis, yet limitations on in vitro expansion of AT2s have hindered the advancement of this cell therapy (9–12).

An existing treatment modality for incurable, chronic respiratory diseases is lung transplantation, yet there are not enough suitable organs to meet the transplant need, with the waiting list retaining approximately 4,000 patients since 2011 (13, 14). Lung tissue engineering offers a means to increase the number of available organs for transplant. Progress toward the generation of engineered lungs has been made using decellularization-recellularization approaches, whereby cells are lysed and removed from a native lung to generate a scaffold, which is then repopulated with cells in a process termed recellularization (15–19). However, despite considerable progress, one of the key remaining challenges facing lung tissue engineering has been obtaining sufficient epithelial cell numbers to recellularize the distal alveoli (17, 20).

Despite their proliferative capacity in vivo, it is widely accepted that AT2s cannot be expanded in vitro using standard 2D culture systems (8, 21). Success for AT2 expansion has been reported in 3D organoid cultures in Matrigel discs, which remains the primary method for AT2 culture (1, 22–25). While organoid cultures offer a robust means to study AT2s in vitro, this method does not support expansion of the billions of cells required for lung tissue engineering or cell therapy. A noteworthy method to scale lung organoid cultures has been reported where Matrigel discs are cultured in suspension in a bioreactor; however, the required manual manipulation and the substantial Matrigel variability concerns present challenges for the eventual large-scale manufacturing of cells using this method (20, 26–28).

Herein, we demonstrate the expansion of primary, human AT2s on microcarriers in a scalable bioreactor system. Cells grown using this method could be expanded for 5 passages while retaining a phenotype similar to freshly isolated AT2s and the ability to differentiate into AT1-like cells. We illustrate the criticality of culture medium, substrate, and stiffness in promoting AT2 expansion and phenotype maintenance. We also establish that scale up to 10 L bioreactors enabled the generation of hundreds of billions of AT2s per single organ donor. Importantly, these expanded AT2s engraft and differentiate into AT1-like cells in decellularized porcine lung scaffolds, demonstrating their utility in tissue engineering applications.

## Results

### Establishment of a scalable method to expand AT2s.

**Figure 1:**
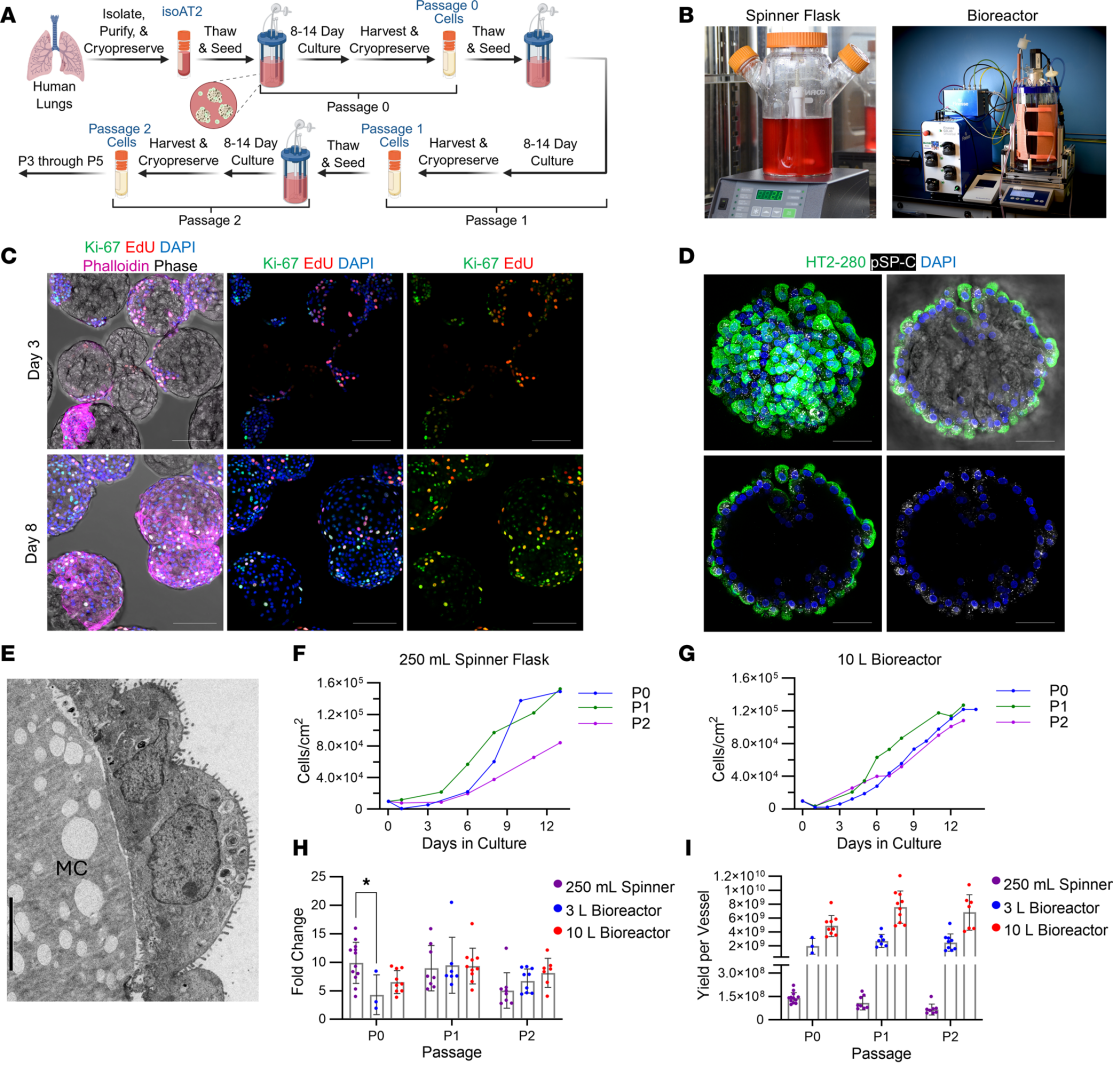
Establishment of a scalable method to expand AT2s. (**A**) Schematic of AT2 expansion. (**B**) Images of the culture systems used to expand AT2s: 250 mL spinner flask and 10 L bioreactor and controller. (**C**) Immunostaining for Ki-67 (green) alongside EdU (red), DAPI (blue), and phalloidin (magenta) on days 3 and 8 of P3 AT2s on microcarriers in T2-Max. Scale bar: 100 μm. (**D**) Immunostaining for AT2-specific proteins on AT2s on microcarriers on day 11 of P3: HT2-280 (green), pSP-C (white), DAPI (blue). Top left: maximum projection; top right: microcarrier cross-section, bright-field merge; lower left: microcarrier cross-section, without bright-field merge; lower right: microcarrier cross-section with pSP-C and DAPI only. Scale bars: 50 μm. (**E**) Transmission electron microscopy image of AT2s on a microcarrier (MC). Scale bar: 5 μm. (**F**) Growth curves from 3 successive cultures of AT2s isolated from donor 9VX and grown in 250 mL spinner flasks. (**G**) Growth curves from 3 successive cultures of AT2s isolated from donor 9VX and grown in 10 L bioreactors. (**H**) Fold change for P0 through P2 at 3 different scales (250 mL spinner, 3L bioreactor, and 10 L bioreactor), with each data point representing fold change for a different donor-derived cell bank. *P < 0.05, mixed effects analysis, Tukey’s multiple comparisons test. (**I**) Cell yields from 250 mL spinner flask, 3 L bioreactor, and 10 L bioreactor scales across 3 passages, with each data point representing yield from a different donor-derived cell bank. All bars represent mean ± SD. In **H** and **I**, 250 mL spinner: *n* = 12 donors, 3 L bioreactor: *n* = 9 donors, 10 L bioreactor: *n* = 10 donors.

The discovery that both mouse and human AT2s can be grown as organoids has enabled widespread study of this unique cell type, yet scalability of the organoid culture platform is limited (1, 22). To achieve cell yields necessary for cell therapy and lung tissue engineering, we developed a scalable method for expanding AT2s, comprising an expansion medium (T2-Max), a gelatin microcarrier culture substrate, and a spinner flask or stirred tank bioreactor culture system (Figure 1A). Briefly, cells were seeded onto microcarriers, and the cell-laden microcarriers were kept in suspension via the rotating impeller of the culture system. Cells can be grown on microcarriers in spinner flasks or bioreactors at different culture scales depending on the starting number of isolated AT2s (Figure 1B). Spinner flask cultures (250 mL) were used as a small-scale method development platform owing to the low cell input and equipment requirements. However, the use of larger scale bioreactors proved critical to meet the required cell yield for organ recellularization. Bioreactor vessels ranged from 3.75 L to 10 L and enabled real-time monitoring and control of culture conditions. While this paper focuses on the microcarrier platform, T2-Max can also support the formation of feeder-free organoids in Matrigel, demonstrating potential applicability to existing AT2 culture platforms (Supplemental Figure 1A; supplemental material available online with this article; https://doi.org/10.1172/JCI188701DS1).

Following attachment, cells proliferated to cover the gelatin microcarriers over the course of an 8- to 14-day culture. Maintenance of proliferation and AT2 phenotype throughout the culture duration and across multiple passages were important considerations during the development of this process. High levels of EdU incorporation and Ki-67 positivity were observed at both early and late time points of culture, days 3 and 8, respectively (Figure 1C). The cells displayed typical cuboidal morphology and stained positive for apical-localized HT2-280 and punctate prosurfactant protein C (pSP-C), two canonical AT2 markers, while growing on microcarriers (Figure 1D and Supplemental Figure 1B). Furthermore, AT2s on microcarriers exhibited microvilli and lamellar bodies (Figure 1E). Upon reaching confluence, dissociation of cells from the microcarriers enabled serial passaging. Screening studies of isolated AT2s revealed that expansion potential is a cell bank–specific variable influenced by donor, tissue handling, and processing. Therefore, we implemented a proliferation assay to identify cell banks likely to fail in the first passage, termed passage 0 (P0; Supplemental Figure 1C). Cell banks with EdU labeling below 40% were not expanded, and data from those cell banks are not further represented in this paper.

Development of the expansion process was aided by daily culture sampling to determine cell concentration and evaluate the culture environment, including pH, dissolved oxygen, and metabolites. AT2s were seeded at a density of 10,000 cells/cm^2^, and cell density typically increased to 80,000–160,000 cells/cm^2^ by the end of each passage, with some cultures exceeding 200,000 cells/cm^2^. Donor-matched expansion over 3 passages at our smallest and largest scales (250 mL vs. 10 L, respectively) demonstrated that both spinner flask and bioreactor culture platforms could be used to expand AT2s, albeit differences exist in the growth rate and final confluence achieved (Figure 1, F and G). Combined data from multiple donors revealed an average fold increase of 7.6 across passage and scale, with significant differences across scale observed only at P0 (Figure 1H). Cell yields depended on culture scale, and on average, the yield was 1.04 × 10^8^ cells from a 250 mL spinner flask (200 mL working volume), 2.24 × 10^9^ cells from a 3 L bioreactor (3.75 L working volume), and 6.43 × 10^9^ cells from a 10 L bioreactor (Figure 1I). To date, AT2s isolated from 3 lung donors were grown to near maximum capacity for 3–4 passages using the expansion method described herein. AT2s isolated from donors R0G, D7V, and 9VX yielded 180 × 10^9^, 337 × 10^9^, 699 × 10^9^ expanded cells, respectively. These are the highest known reported yields of primary human AT2s. Upon exhaustion of the banks, cell yields from donor D7V are expected to surpass 1 × 10^12^ expanded AT2s at P2 or P3.

An extended passaging study was performed to determine if AT2s could be grown beyond P3 using this expansion method. AT2s isolated from 4 donors expanded for at least 5–6 passages in bioreactors or a total of 59–76 culture days. Three of the four cell banks reached a culture population doubling level (PDL) of 30–31, and their proliferative capacity decreased only modestly through P6, with culture population doubling time (PDT) maintained within a 2-fold increase of the P0 PDT for each bank (Supplemental Figure 1, D and E). The fourth cell bank reached a culture PDL of 23, and the AT2s surpassed a 2-fold increase in culture PDT during the P4 culture. While donor heterogeneity influenced the number of passages a given cell bank could be expanded, these results demonstrated the vast expansion potential of cells grown using this method. Finally, karyotyping was performed on 34 samples from 7 donors across multiple passages to confirm that this expansion method does not drive genomic instability. Results revealed that expanded AT2s were fairly stable, with only 2 abnormalities in a low number of cells (2 of 20) that did not persist over passage and nonclonal findings that did not repeat or increase in number with passage (Supplemental Figure 1F). These data suggest the changes are likely random errors in cell division and are not specific to the expansion method.

### AT2 phenotype after expansion is largely similar to that of freshly isolated AT2s.

**Figure 2:**
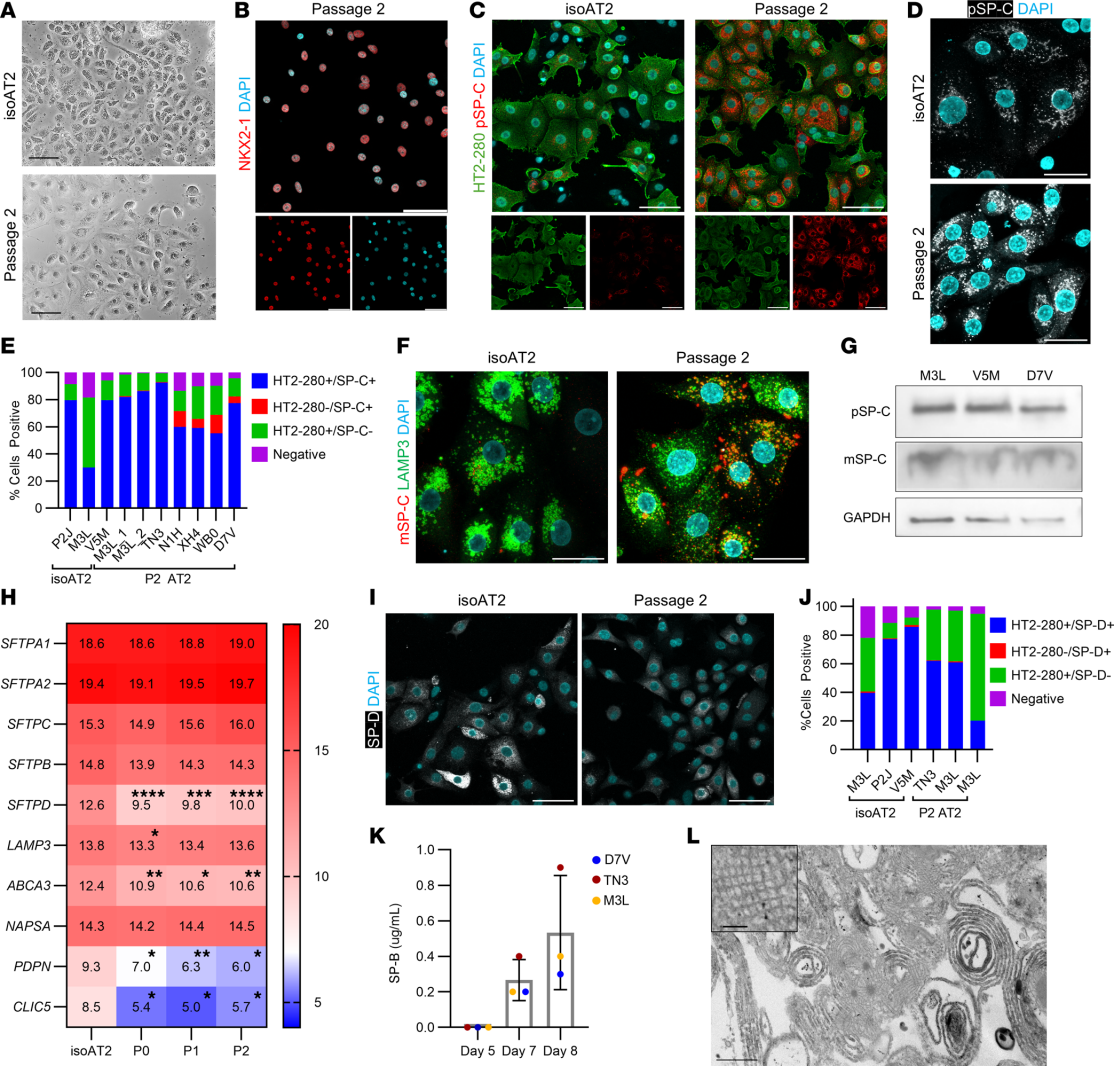
AT2 phenotype after expansion is largely similar to freshly isolated AT2s. (**A**) Bright-field images comparing isoAT2s to P2 AT2s. Scale bar: 100 μm. (**B**) Immunostaining for NKX2-1 (red) in P2 AT2s. Scale bar: 50 μm (**C**) Immunostaining for HT2-280 (green) and pSP-C (red) in isoAT2s and P2 AT2s. Scale bar: 50 μm. (**D**) Immunostaining for pSP-C (gray) in isoAT2s and P2 AT2s at higher magnification. Scale bar: 25 μm. (**E**) Quantitation of HT2-280 and pSP-C as a percentage of cells expressing both, none, or either protein above secondary-only control (isoAT2, *n* = 3 donors; P2 AT2, *n* = 6 donors; mean of *n* = 3 wells per bank; unpaired *t* test of HT2-280^+^/pSP-C^+^ population in P2 AT2 compared with isoAT2, *P* = 0.22). (**F**) Immunostaining for LAMP3 (green) and mSP-C (red) in isoAT2s and P2 AT2s. Scale bar: 25 μm. (**G**) Western blot of pSP-C and mSP-C in P2 AT2 lysates (*n* = 3 donors). (**H**) Quantitation of bulk mRNA transcripts in isoAT2s and expanded AT2s (*n* = 6 donors per passage; mean of log_2_ normalized counts; repeated measures 1-way ANOVA, Tukey’s multiple comparisons test, *P < 0.05, **P < 0.01, ***P < 0.001, ****P < 0.0001 compared with isoAT2s). (**I**) Immunostaining for SP-D (gray) in isoAT2s and P2 AT2s. Scale bar: 50 μm. (**J**) Quantitation of HT2-280 and SP-D immunostaining as a percentage of cells expressing both, none, or either protein above secondary-only control (*n* = 3 AT2 banks/passage; mean of *n* = 6 wells per bank; unpaired *t* test of HT2-280/SPD dual-positive population in P2 AT2 compared with isoAT2, *P* = 0.38). (**K**) HPLC analysis of SP-B in AT2-conditioned media collected from bioreactors after day 5 media exchange (P3; *n* = 3 donors). Data represent mean ± SD. (**L**) Transmission electron microscopy images showing structures resembling tubular myelin in conditioned media. Scale bar: 0.5 μm (main image); 0.1 μm (top left). DAPI (cyan) was used as a counterstain in **B**, **C**, **D**, **F** and **I**. Scale bar: 50 μm, unless otherwise noted.

Following harvest from microcarrier cultures, expanded AT2s displayed similar morphology to freshly isolated, nonexpanded AT2s (referred to herein as isoAT2s), exhibiting characteristic epithelial size and shape (Figure 2A). Expression of NKX2-1, a transcription factor specific to lung identity, and lack of expression of nonalveolar identity markers were confirmed via immunostaining (Figure 2B and Supplemental Figure 2, A and B). HT2-280 and SP-C are markers used to identify AT2s (29). Both were expressed on a majority of AT2s after expansion on microcarriers, with SP-C evident as both newly synthesized pSP-C and mature-SP-C (mSP-C), which was frequently colocalized with the lamellar body–associated protein, LAMP3 (Figure 2, C–F and Supplemental Figure 2C). Qualitative assessment revealed more SP-C per cell in P2 AT2s vs. isoAT2s, yet quantification demonstrated no significant difference in the HT2-280/pSP-C dual-positive population for expanded AT2s versus isoAT2s (Figure 2E). We hypothesize the intensity difference is related to the need for cell recovery following the isolation process. Heterogeneity in the proportion of dual-positive cells was evident across samples and may be a donor-linked variable. In the extended passage study (Supplemental Figure 1), the AT2 population was maintained through P4 or P5, where expanded AT2s existed as HT2-280–positive, pSP-C–positive, or dual-positive, with minimal expression of the basal cell markers, CK5 and p63 (Supplemental Figure 2, E–G). While the culture still largely consisted of epithelial cells at P6, exhibited by 99% EpCAM expression, the AT2 population began to decrease, and basal cell markers began to increase. Therefore, we recommend terminating AT2 cultures after P4 or P5, depending on individual bank performance (Supplemental Figure 2, D and H).

We next evaluated AT2-specific gene expression in expanded cells versus isoAT2s using mRNA profiling. Several AT2 markers (*SFTPA1*, *SFTPA2*, *SFTPC, SFTPB*, and *NAPSA*) were not significantly changed in expanded AT2s, while *LAMP3* was significantly reduced only at P0, and *SFTPD* and *ABCA3* were significantly reduced at all passages (Figure 2H). The AT1 markers *PDPN* and *CLIC5* decreased in P0 and remained at the limit of detection. Despite reduced gene expression, quantitative immunostaining for SP-D revealed that protein expression in expanded AT2s was not significantly different from that of isoAT2s owing to overall heterogeneity across donors and banks (Figure 2, I and J). We also evaluated expanded AT2s for transitional markers and observed minimal protein expression by immunostaining and mostly stable gene expression (Supplemental Figure 2, I and J). An aberrant basaloid marker, *KRT17*, increased in culture, albeit not significantly. Significant gene upregulation was only observed for *TGFB2* and *TP53* at P2 and P0, respectively, suggesting that expansion led to changes but did not largely elicit a transitional state (Supplemental Figure 2J) (30–33). A key functional role of AT2s is surfactant secretion, which we confirmed through high-performance liquid chromatography evaluation of SP-B and transmission electron microscopy of the conditioned media collected from bioreactors (Figure 2, K and L). Taken together, it is evident that, despite some changes following culture, expanded AT2s largely maintained key characteristics of their native counterparts.

### Single-cell RNA-seq confirms expected AT2 profile after expansion.

**Figure 3:**
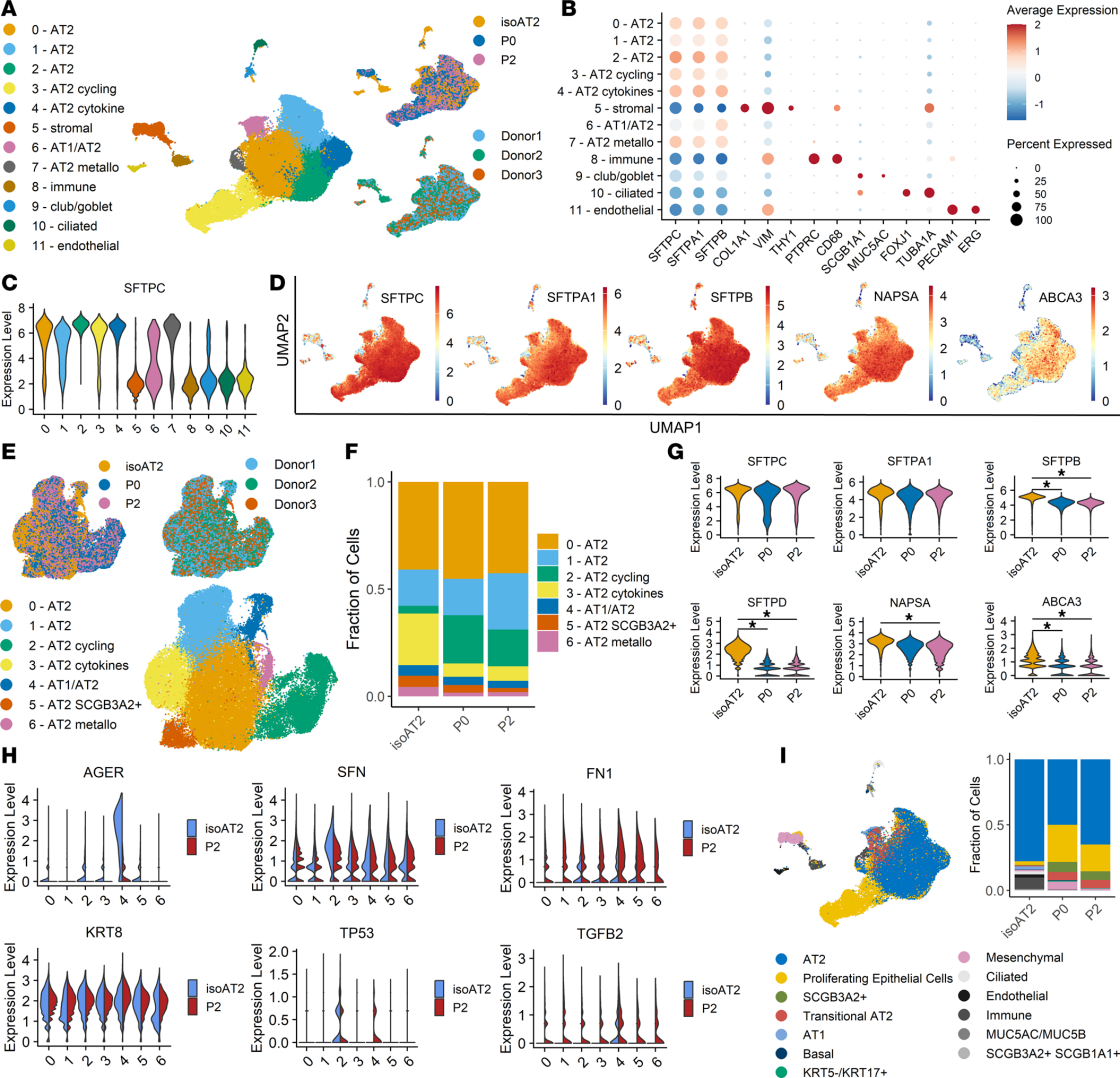
Single-cell RNA-seq confirms expected AT2 profile after expansion. (**A**) UMAP of isoAT2s, P0 AT2s, and P2 AT2s from 3 donors by cell type, sample type, and donor. (**B**) Dot plot of marker expression by cluster. (**C**) Violin plot of *SFTPC* expression in each cluster. (**D**) UMAP of AT2 marker gene expression. (**E**) UMAP of alveolar clusters only, by cell type, sample type, and donor. (**F**) Proportion plot of alveolar clusters comparing isoAT2s, P0 AT2s, and P2 AT2s. (**G**) Violin plots of AT2 marker genes comparing isoAT2s, P0 AT2s, and P2 AT2s (generalized linear model quasi-likelihood F test on sample pseudobulk expressions, FDR < 0.05). (**H**) Split violin plots of known transitional markers. (**I**) UMAP and proportion plot of isoAT2, P0, and P2 cells identified using reference-based label-transfer cell typing from Habermann et al. (37).

To further interrogate the expanded AT2 phenotype, single-cell RNA-seq (scRNA-seq) was performed on isoAT2s, P0 AT2s, and P2 AT2s from 3 donors. Map-based clustering and canonical marker cell typing confirmed that a majority of the cells were of lung origin and, more specifically, were AT2s (Figure 3, A–D, and Supplemental Figure 3, A–H). There were small populations of stromal, conducting airway, endothelial, and immune cells in the isoAT2 samples that diminished with expansion. Reclustering just the alveolar cells revealed a large cluster (named cluster 0) with typical AT2 gene expression and 6 additional subclusters with unique marker enrichment, most of which have been described previously (Figure 3E and Supplemental Figure 3, I–N, and Supplemental Table 1) (34–36). Lesser-known alveolar populations included cluster 1, characterized by slightly diminished AT2 gene expression, increased mitochondrial gene expression, and increased expression of long noncoding RNAs, *MALAT1* and *NEAT1*, and cluster 6, marked by upregulation of metallothionein genes. When aggregating cells in clusters 0 or 1, no differentially expressed genes between clusters were identified, and we therefore simply refer to these cells by their cluster numbers. The most notable shift in the alveolar population during expansion was an increase in the cycling population and a corresponding decrease in the cytokine-responsive population (Figure 3F). These proportion shifts were also mirrored in differential gene expression and pathway enrichment analysis, whereby interleukin signaling was prevalent in isoAT2s, and cell cycle pathways were enriched in P2 AT2s (Supplemental Figure 3, O and P, and Supplemental Table 2). Reviewing canonical AT2 markers over passage demonstrated overall maintenance of AT2 gene expression and matched the bulk RNA results described herein, with *SFTPD* and *ABCA3* as the exceptions (Figure 3G). There was no increase in the proportion of the AT1/2 cluster, characterized by diminished AT2 marker expression and increased AT1 marker expression, over passage. Probing into reported human AT2 transitional markers and aberrant basaloid markers across all clusters comparing isoAT2s to P2 AT2s revealed increases in individual genes that were not restricted to a single cluster (*FN1, TGFB2, KRT17*) and a lack of upregulation of core aberrant disease-associated markers (*CDH2, COL1A1, SERPINE1, EPHB2*) (Figure 3H, Supplemental Figure 3Q, and Supplemental Figure 4) (37, 38). Taken together, these analyses further confirmed that the expanded cells did not exhibit a clear transitional or aberrant cell signature (33, 37, 39, 40). Reference-based label-transfer cell typing using the dataset generated by Habermann et al. was performed, and the assigned labels were mapped onto the original UMAP embedding (37). Alignment with the map-based clustering was evident, and only a modest increase in the transitional population during expansion was observed (Figure 3I). Furthermore, the *KRT5^–^KRT17^+^* aberrant transitional cells associated with lung disease only represented 0.28% of our P2 AT2s (Figure 3I) (37). Collectively, these sequencing results underscore that our expansion method maintains normal AT2s.

### Three exogenous factors enable the expansion and stability of primary AT2s in vitro.

Standard 2D culture methods are insufficient for expanding AT2s owing to loss of phenotype (8, 21, 41). Our own attempts to expand AT2s in collagen-coated flasks revealed a nearly complete loss of pSP-C at P0 as well as a diminishing population of HT2-280–positive cells, despite their epithelial appearance and retention of EpCAM expression (Supplemental Figure 5, A and B). As such, organoid-based culture platforms using specialized media are the prevailing method for growing AT2s ex vivo, with exogenous signaling factors in the media used as the primary means to control AT2 fate (1, 3, 42). Furthermore, Matrigel is commonly utilized as a substrate for alveolar organoids since it provides a mixture of ECM components and has an elastic modulus in the same range as lung parenchyma (42, 43).

**Figure 4:**
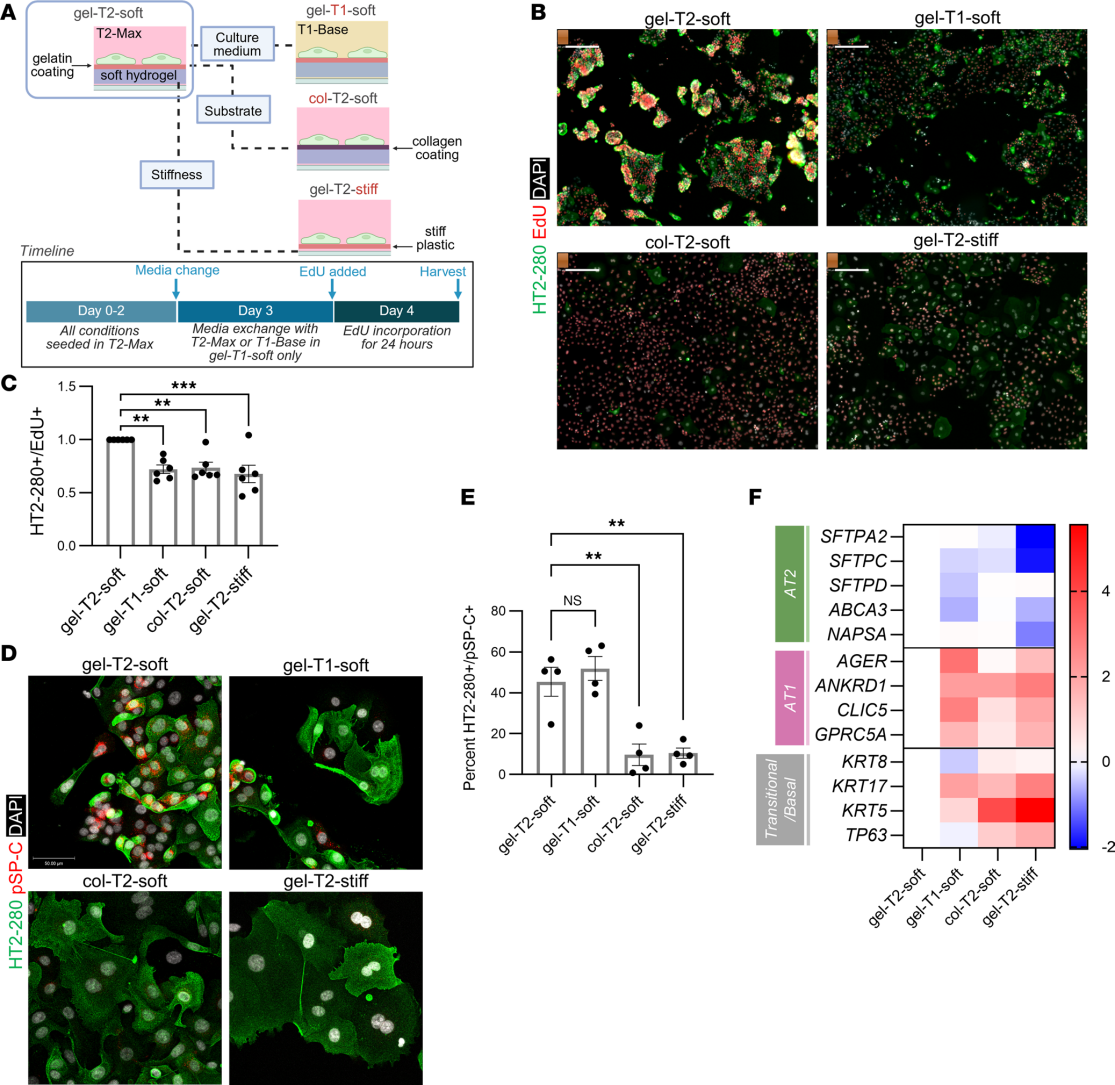
Three exogenous factors enable the expansion and stability of primary AT2s in vitro. (**A**) Schematic of conditions and experimental timeline. (**B**) Representative immunostaining of HT2-280 (green) alongside EdU (red) in P0 AT2s across each condition. Coatings: gelatin (gel) or collagen (col). Media: T2-Max (T2) or T1-Base (T1). Stiffness: 12-16 kPa (soft) or approximately 1 GPa (stiff). Scale bar: 200 μm. (**C**) Quantitation of cells positive for both HT2-280 and EdU as a fraction of total nucleated cells (mean ± SEM, *n* = 6 donors, 2 experiments). Normalized to gel-T2-soft condition; 1-way ANOVA, Dunnett’s post hoc test, **P < 0.01, ***P < 0.001. (**D**) Immunostaining of HT2-280 (green) and pSP-C (red) across each condition. Scale bar: 50 μm. (**E**) Quantitation of cells expressing HT2-280 and pSP-C across each media/substrate condition (mean ± SEM, *n* = 4 donors, 1 experiment). One-way ANOVA, Dunnett’s post hoc test, **P < 0.01. (**F**) Quantitation of gene expression using qRT-PCR (log_2_ fold change) relative to gel-T2-soft control. Genes are clustered by cell type: AT2, AT1, and transitional/basal. DAPI (cyan) was used as a counterstain in **B** and **D**.

To elucidate the supportive mechanism behind our culture platform, 2D cultures were performed to isolate the effects of 3 critical factors: substrate composition, stiffness (elastic modulus), and media formulation. We hypothesized that all 3 factors synergistically support the proliferation and phenotype of AT2s during ex vivo expansion. The microcarriers used herein were composed of gelatin and have a low elastic modulus of approximately 16 kPa, within the range of lung parenchyma. To closely match these microcarrier properties, commercially available tissue culture plates with a coated hydrogel surface and a defined stiffness were utilized. The control condition was gelatin coating in T2-Max on a substrate with low elastic modulus (termed gel-T2-soft to identify substrate-media-stiffness conditions). Gel-T2-soft was compared with test conditions that systematically altered 1 of the 3 key variables as follows: (a) gel-T1-soft, T1-Base in place of T2-Max, which is the same base medium formulation as T2-Max with all growth factors and small molecules removed; (b) col-T2-soft, type 1 collagen in place of gelatin; and (c) gel-T2-stiff, a polystyrene plate in place of the hydrogel (Figure 4A). Click-iT EdU labeling was multiplexed with HT2-280 immunostaining to quantify proliferation specifically in AT2s.

All test conditions exhibited a significantly reduced HT2-280 and EdU dual-positive fraction compared with control (Figure 4, B and C). This reduction in proliferating AT2s was paired with a significant increase in the HT2-280–negative, EdU-positive fraction for col-T2-soft and gel-T2-stiff conditions (Supplemental Figure 5C). pSP-C protein expression was also significantly reduced in col-T2-soft and gel-T2-stiff conditions but not in gel-T1-soft, likely due to the short culture time in T1-Base media (Figure 4, D and E). To further elucidate the effect of substrate stiffness on AT2 phenotype, hydrogel plates with a modular stiffness ranging from 0.1 to 100 kPa were employed. Cell morphology gradually shifted from spheroidal to flattened when comparing 0.1 kPa with 100 kPa (Supplemental Figure 6A). Furthermore, HT2-280 and pSP-C staining decreased as stiffness increased (Supplemental Figure 6, B–D). In alignment with the mechanism study (Figure 4), AT2 phenotype was better maintained on gelatin-coated hydrogels compared with collagen-coated hydrogels. Analysis of mRNA expression indicated altering any 1 of the 3 critical factors (substrate, culture medium, or stiffness) promoted differentiation, where AT1 markers (*AGER*, *ANKRD1*, *CLIC5*, *GPRC5A*) and transitional/basal markers (*KRT8, KRT17, KRT5, TP63*) were upregulated in each test condition (Figure 4F). Collectively, these data demonstrate the importance of substrate composition, substrate stiffness, and media formulation for the successful maintenance of AT2 phenotype and proliferative capacity in our large-scale expansion method.

### Expanded AT2s express AT1 markers after culture in AT1 medium.

A critical AT2 progenitor cell responsibility is differentiation into AT1s in response to lung injury to repair the alveolar epithelium (1, 5, 44, 45). Expanded AT2s were evaluated for their ability to differentiate into cells expressing molecular and behavioral characteristics of AT1s. For this purpose, an alveolar differentiation medium, T1-Diff, was established. While the precise mechanisms that drive AT2-to-AT1 differentiation in human adult lungs are still under active investigation, several relevant signaling pathways involved in differentiation have been identified, including Wnt/β-catenin, Etv5, BMP, Notch, TGF-β, and YAP/TAZ (44). LATS inhibition was recently shown to robustly induce AT2-to-AT1 differentiation by driving nuclear YAP activation (40, 46). Therefore, to generate AT1 differentiation medium (T1-Diff), all media additives in T2-Max that promoted AT2 proliferation and phenotype maintenance were excluded and replaced with a LATS inhibitor. The only difference between T1-Diff and T1-Base is the addition of the LATS inhibitor to T1-Base to generate T1-Diff.

**Figure 5:**
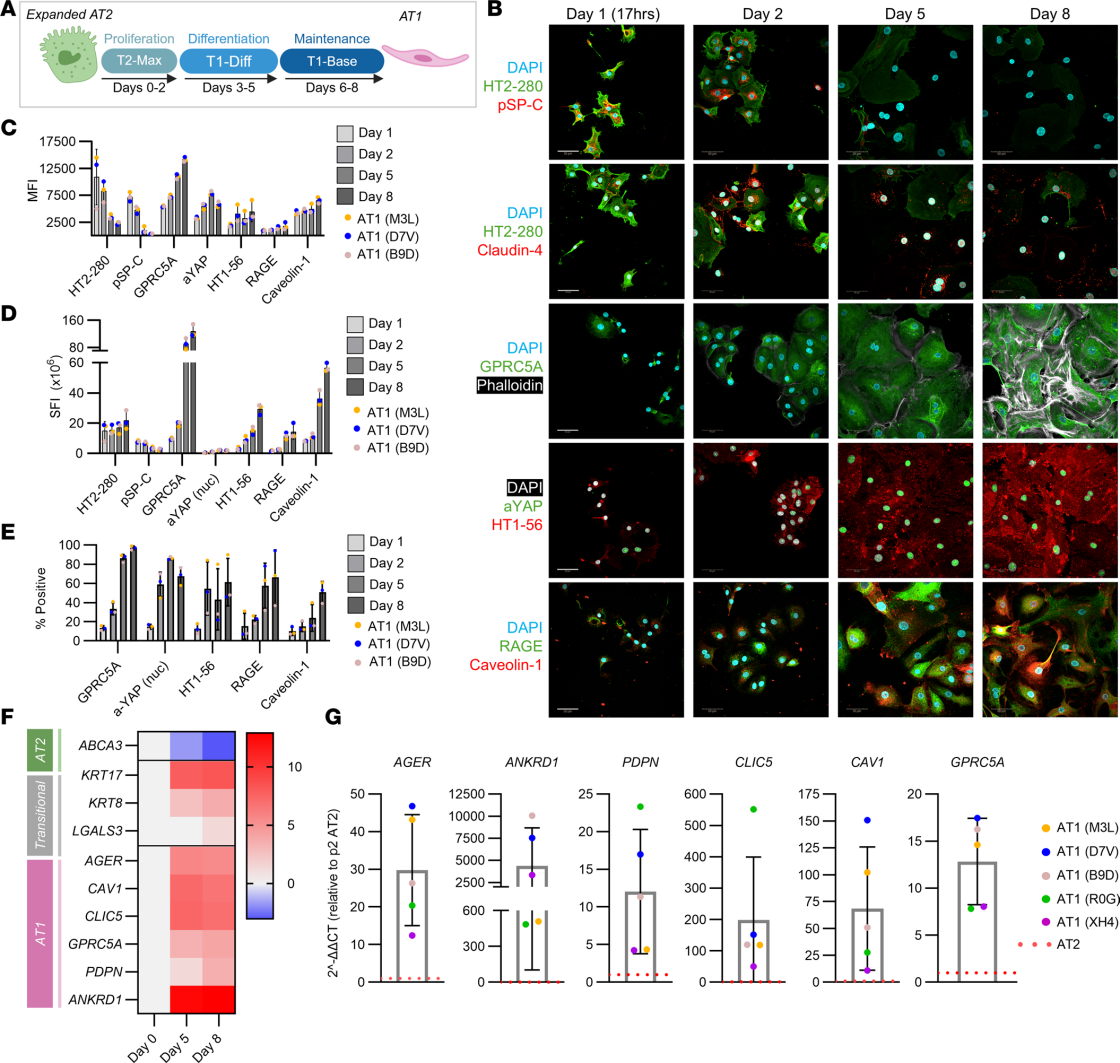
Expanded AT2s express AT1 markers after culture in AT1 medium. (**A**) Schematic representation of AT2-to-AT1 differentiation. Expanded AT2s were cultured in T2-Max for 2 days, T1-Diff for 3 days, and T1-Base for 3 days to generate AT1s. (**B**) Immunostaining time course from the AT1 differentiation paradigm described in **A**. Scale bars: 50 μm. (**C**) Immunostaining quantification of AT1 differentiation. Mean fluorescent intensity (MFI) of cells expressing each target was quantified for cells from each donor on days 1, 2, 5, and 8 (nuclear [nuc]; *n* = 3 donors; mean ± SD). (**D**) Immunostaining quantification of AT1 differentiation. Sum fluorescent intensity (SFI) of cells expressing each target was quantified for cells from each donor on days 1, 2, 5, and 8 (*n* = 3 donors; mean ± SD). (**E**) Immunostaining quantification of AT1 differentiation. Quantification of proportion of cells expressing each target above the day 1 MFI+1 SD (*n* = 3 donors; mean ± SD). (**F**) Gene expression quantification using qRT-PCR of AT1 differentiation over time (log_2_ fold change normalized to donor-specific P2 AT2 controls; *n* = 3 donors). (**G**) Gene expression quantification using qRT-PCR of AT1-specific markers on day 8 (2^-ΔΔCT^ normalized to donor-specific P2 AT2 controls (red dotted line); *n* = 5 donors; mean ± SD).

For in vitro assessment of T1-Diff–derived AT1s, expanded AT2s from 3 donors were seeded onto collagen I–coated cultureware and cultured in T2-Max medium for 2 days before being switched to T1-Diff on day 2 to promote differentiation. After 3 days in T1-Diff, cells were cultured in T1-Base for a 3-day maintenance phase, with time-course takedowns on days 1, 2, 5, and 8 (Figure 5A). Immunostaining with quantification of mean fluorescence intensity (MFI) and sum fluorescence intensity (SFI) for AT2, transitional cell, and AT1 markers revealed near-complete loss of HT2-280 and pSP-C expression paired with increased AT1 markers, including GPRC5A, nuclear-localized active YAP (aYAP), HT1-56, RAGE, and caveolin-1 (Figure 5, B–D, and Supplemental Figure 7A) (40, 47). Given the increase in cell size and flattening of the cytosol that occurs during AT1 transition, in some cases, SFI captured the changes in protein expression more readily than MFI. This was particularly evident for caveolin-1, HT1-56, and RAGE. Flattening of the AT1s also revealed a pronounced actin cytoskeletal network visualized via phalloidin staining, which is consistent with reports highlighting the importance of cytoskeletal forces in AT1 shape and function (40, 47, 48). Transitional marker, claudin-4, was predominantly localized to cell junctions in the first 2 time points and diminished over time, consistent with reported transient increases in transitional cells (33). By day 8, a majority of cells were positive for AT1 markers at a higher intensity than the day 1 time point: 97% GPRC5a positive, 68% aYAP positive, 61% HT1-56 positive, 66% RAGE positive, and 51% caveolin-1 positive (Figure 5E).

Furthermore, T1-Diff induced a transcriptomic shift, demonstrated by downregulation of the AT2-specific gene, *ABCA3*, and upregulation of transitional and AT1 genes, *KRT17, KRT8, LGALS3, AGER, CAV1*, *CLIC5, GPRC5A, PDPN,* and *ANKRD1*, on day 5 that remained upregulated through day 8 (Figure 5F). Gene expression analysis of day 8 AT1s generated from 5 donors revealed robust enrichment of AT1 mRNA after differentiation (Figure 5G). Importantly, *CLIC5* and *PDPN* increased as expected, despite the aforementioned lack of increase in protein expression. Differences in AT1 gene enhancement across the 5 banks were associated with variability in AT1 gene expression in P2 AT2s, before initiating AT1 conversion. P2 banks with relatively higher AT1 gene expression immediately following expansion exhibited lower overall increases in AT1-specific genes following media-driven conversion (Supplemental Figure 7B). Taken together, primary human AT2s retain the capacity to express AT1 markers following large-scale expansion. Upregulation of transitional cell markers on day 8 brings about the possibility that differentiation may be incomplete and may require more time or culture in a more biomimetic platform, such as a lung scaffold.

To confirm LATS inhibition and subsequent nuclear aYAP expression are responsible for driving differentiation of expanded AT2s into AT1s, gene expression of cells cultured in T1-Diff and T1-Base was assessed. Cells cultured in T1-Diff and T1-Base both exhibited robust upregulation of AT1 genes, *AGER*, *ANKRD1, GPRC5A*, *CLIC5*, *PDPN*, and *CAV1*, with a trend toward higher expression with the LATS inhibitor that was not statistically significant (Supplemental Figure 7C).

### AT1s derived from expanded AT2s exhibit key characteristics.

**Figure 6:**
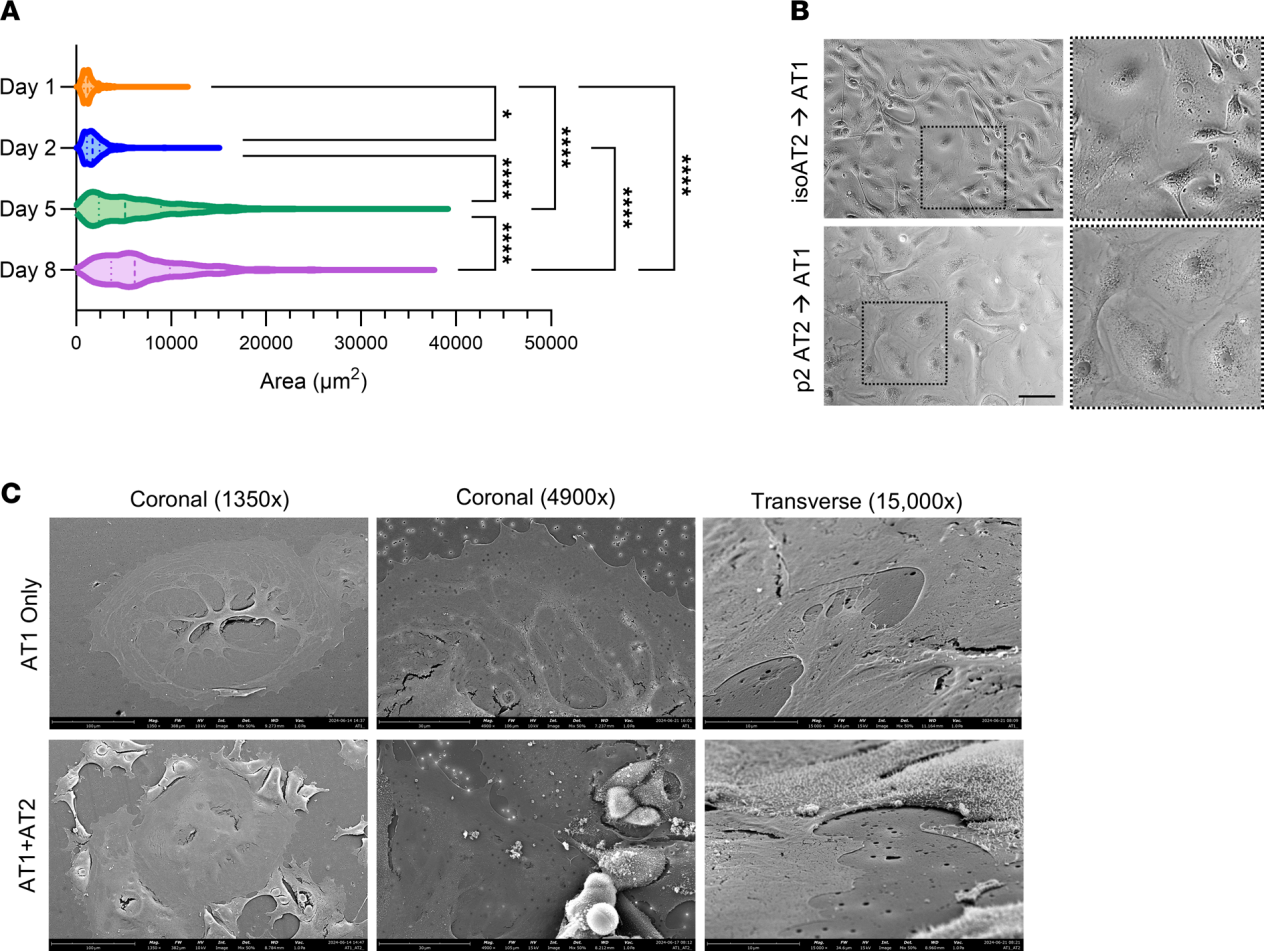
AT1s derived from expanded AT2s exhibit morphological changes. (**A**) Violin plot of cell area quantification during AT2-to-AT1 differentiation. Analysis was performed on 3 donors after 17 hours in T2-Max (day 1, *n* = 534 cells), prior to switching to T1-Diff on day 2 (*n* = 1091 cells), prior to switching to T1-Base on day 5 (*n* = 1647 cells), and at completion of culture on day 8 (*n* = 936 cells; 1-way ANOVA with Tukey’s multiple comparison test; *P = 0.0219, ****P < 0.0001). Data represent mean ± SD. AT1s were derived from expanded AT2s. (**B**) Bright-field images with insets of AT1s derived from isoAT2s and expanded AT2s. Scale bar: 100 μm. (**C**) Scanning electron microscopy images of AT1s cultured on Transwell inserts alone (AT1 only) or with a second late seed of expanded AT2s for comparison (AT1+AT2). Images were taken from above (coronal) and at an angle (transverse). AT1s were derived from expanded AT2s.

AT1s are remarkably large, thin cells that cover approximately 95% of the alveolar surface and are closely aligned with the capillary network of the lung to facilitate passive gas exchange (7, 49–51). The sizes of expanded AT2s taken through the AT1 differentiation paradigm were quantified over time and revealed a 461% increase in cell area from day 1 to day 5, which further increased through day 8, after removal of the LATS inhibitor (Figure 6A). Furthermore, cell area following differentiation was similar to the reported average surface area of human adult AT1s (measured average, 7,296 μm^2^ vs. reported average, 5,100 μm^2^) (7, 50). The size of the AT1s derived from expanded AT2s was qualitatively compared with that of AT1s derived from isoAT2s that did not go through expansion and was found to be comparable (Figure 6B).

Another hallmark characteristic of AT1s is cell thickness. With a reported value of only 0.1 μm, the AT1 allows for efficient gas exchange across the cell membrane and passive diffusion of oxygen into the bloodstream (50–52). The thickness of AT1-like cells generated from expanded AT2s was visualized on Transwell inserts using a scanning electron microscope. A subset of AT1 cultures received a second AT2 seed the day before fixation for comparison. Imaging revealed hallmark features of AT1s, including a flattened, elongated nucleus; loss of microvilli; and extraordinarily thin, cytoplasmic extensions in comparison to AT2s (Figure 6C) (50).

**Figure 7:**
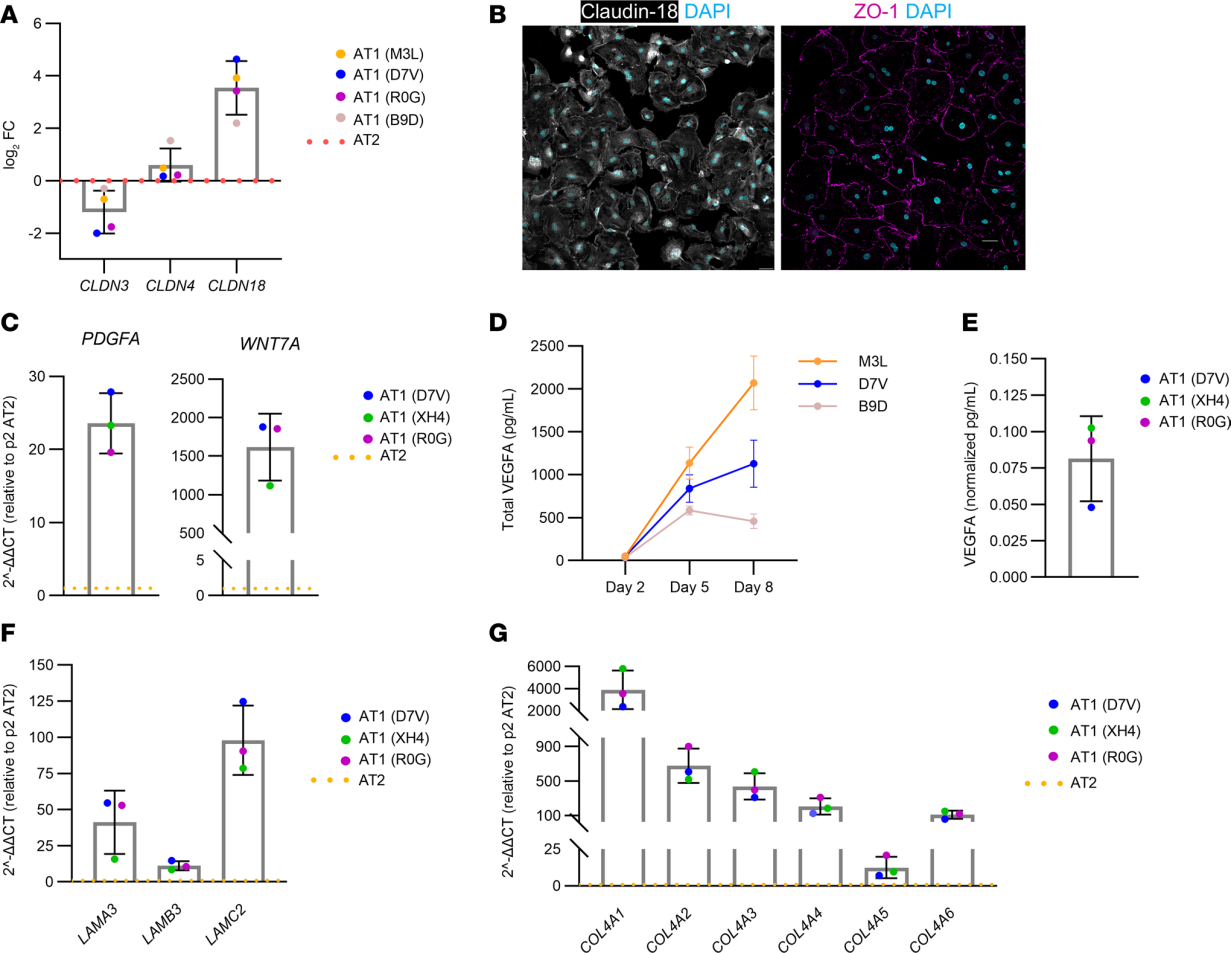
AT1s derived from expanded AT2s exhibit key characteristics. (**A**) Gene expression quantification using qRT-PCR of alveolar-specific claudins from AT1s (log_2_ fold change normalized to donor-specific p2 AT2s controls [red dotted line]; *n* = 4 donors). (**B**) Immunostaining of AT1s for claudin-18 (white), ZO-1 (fuchsia), and DAPI (cyan). Scale bars: 50 μm. (**C**) Gene expression quantification using qRT-PCR of secretory ligands from AT1s (2^-ΔΔCT normalized to donor-specific p2 AT2 controls (orange dotted line); *n* = 3 donors). (**D**) Total VEGF-A protein quantitation in conditioned media samples collected from AT1s on days 2, 5, and 8 (*n* = 3 donors; mean ± SD). (**E**) VEGF-A protein quantitation in conditioned media samples collected from day 8 AT1s. Concentrations normalized to nuclear cell counts from a satellite culture at day 8 (*n* = 3 donors; 3 independent experiments). (**F** and **G**) Gene expression quantification using qRT-PCR of ECM genes from AT1s (2^-ΔΔCT^ normalized to donor-specific p2 AT2s controls (orange dotted line); *n* = 3 donors). All cells analyzed were expanded AT2-derived AT1s. Data represent mean ± SD.

AT1s also aid in maintaining the epithelial barrier and paracellular transport of water and solutes through cell type–specific tight junction complexes composed of various occludins and claudins (53–56). Both AT1s and AT2s express claudin-3, claudin-4, and claudin-18, but their expression patterns differ (54, 56). Claudin-3 is the predominant claudin expressed in AT2s, and claudin-18 is the predominant claudin expressed in AT1s (53, 56, 57). Gene expression analysis of AT1s generated from expanded AT2s from 4 donors revealed an average 3.5-fold upregulation of *CLDN18* and an average 1.2-fold downregulation of *CLDN3* compared with the starting population of AT2s, which aligns with previously published claudin profiles in alveolar tissue (Figure 7A) (56). Protein expression and junctional localization of claudin-18 and ZO-1 were confirmed in our AT1s, demonstrating their capacity to form tight junctions (Figure 7B).

Paracrine signaling from AT1s in the alveolar niche was recently identified as a critical function during alveologenesis and homeostasis in both mouse and human lungs (58). Enriched expression of the genes encoding signaling ligands, *VEGFA*, *PDGFA*, *SHH*, and *WNT7A*, has been demonstrated in both human primary and iPS-derived AT1s (40, 58). Gene expression analysis of our day 8 AT1-like cells generated from 3 donors revealed robust enrichment of AT1 secretory ligand mRNA after differentiation, with an average 24-fold increase in *PDGFA* expression and an average 1,616-fold increase in *WNT7A* (Figure 7C). Furthermore, recent work using transgenic conditional knockout and reporter mouse models demonstrated that VEGF-A is predominantly expressed by AT1s, rather than AT2s, as originally thought; VEGF-A is required locally to promote alveologenesis (59). VEGF secretion by our AT1s was of particular interest given the intended use of these cells in lung tissue engineering, as we hypothesize that AT1-secreted VEGF could aid in recruiting endothelial cells to distal vascular spaces. Conditioned media was collected from expanded AT2 cultures taken through AT1 differentiation and assayed for VEGF-A using an ELISA. While a low concentration of VEGF-A was secreted by expanded AT2s in T2-Max, VEGF-A secretion increased following culture in T1-Diff and T1-Base, reaching an average 27-fold increase over the differentiation time course (Figure 7, D and E).

In addition to playing a key signaling role in the alveolus, AT1s have been recognized as critical in establishing the alveolar basement membrane (40, 60). Consistent with these reports, gene expression analysis of day 8 AT1-like cells generated from 3 donors revealed enrichment of transcripts encoding essential basement membrane proteins, including laminin-332 (*LAMA3*, *LAMB3*, and *LAMC2*) and collagen IV (Figure 7, F and G) (40, 60). In aggregate, these data demonstrate that expanded AT2s retain the capacity to differentiate into AT1-like cells that display hallmark phenotypical and behavioral characteristics of native AT1s.

### Expanded AT2s can sufficiently recellularize porcine scaffolds.

**Figure 8:**
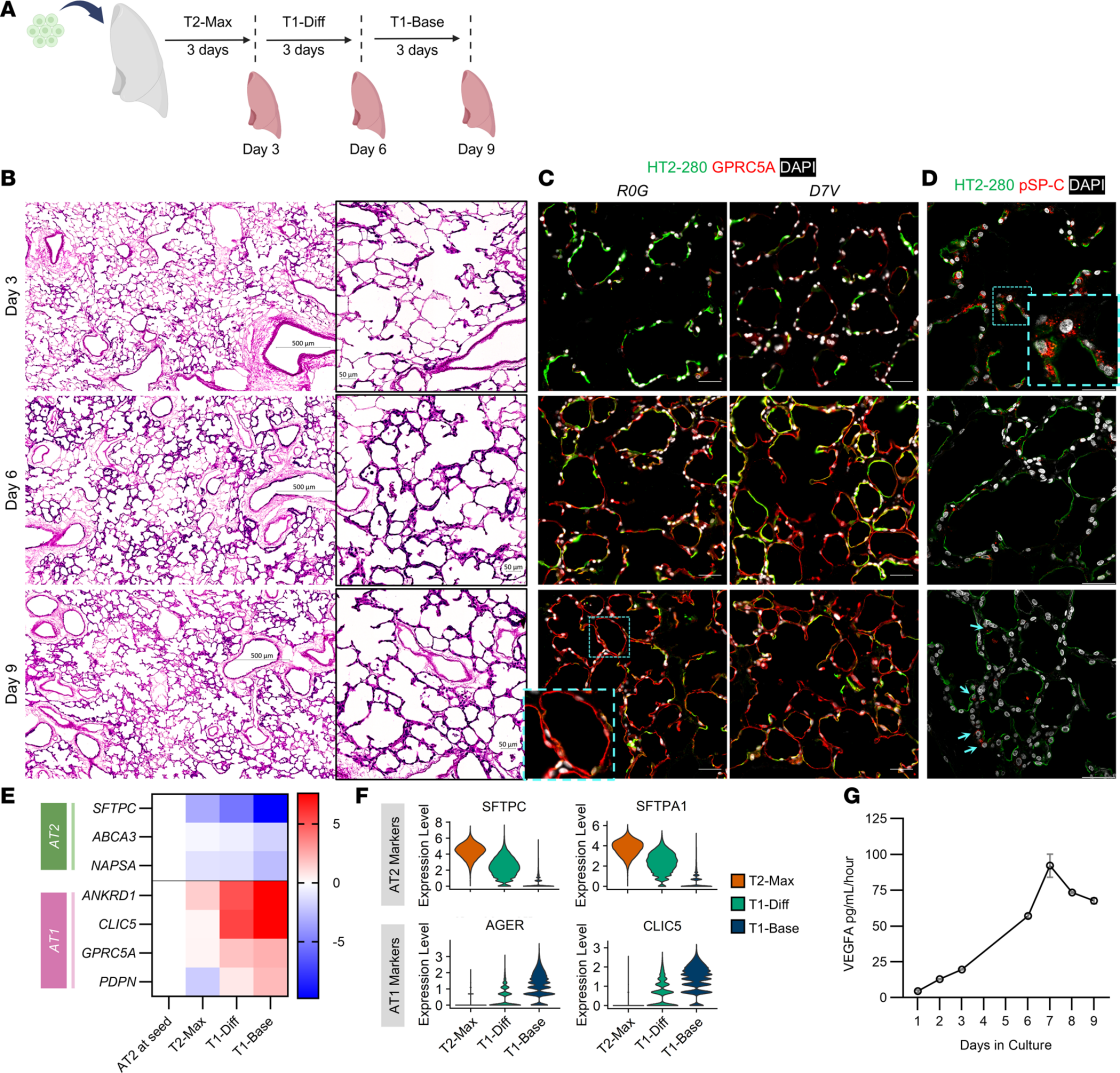
Expanded AT2s can sufficiently recellularize porcine scaffolds. (**A**) Schematic of recellularization process. (**B**) H&E tile scans with zoomed insets from cultures taken down on days 3, 6, and 9. Scale bar: 500 μm (tile scans); 50 μm (insets). (**C**) Immunostaining of HT2-280 (green), GPRC5a (red), and DAPI (white) at each time point across recellularization experiments from 2 AT2 donor banks, with insets showing higher magnification views (cyan box). Scale bars: 50 μm; original magnification, ×20 (insets). (**D**) Immunostaining of HT2-280 (green), pSP-C (red, blue arrows), and DAPI (white) at each time point, with inset (cyan box). Scale bars: 50 μm. (**E**) Gene expression quantitation using qRT-PCR of AT2 markers and AT1 markers at each time point relative to AT2s seeded (log_2_ fold change; *n* = 2 AT2 donor banks per time point). (**F**) snRNA-seq marker expression at each time point across recellularization experiments. (**G**) VEGF-A protein quantitation in conditioned media from recellularized porcine scaffolds (mean ± SD; *n* = 2 donors).

To further demonstrate AT2 capacity to differentiate into AT1s at the tissue engineering scale, a total of 2 × 10^9^ expanded AT2s from 2 unique donors were seeded via airway delivery into decellularized porcine lung scaffolds. T2-Max was utilized from days 0–2 to promote growth and fill in across the alveolar surface before initiating differentiation with a switch to T1-Diff on day 3 and maintenance in T1-Base from day 6 until day 9 (Figure 8A). To examine the distribution and phenotype of AT2s over time, a scaffold from each AT2 donor was analyzed at the end of each media phase. Widespread engraftment of AT2s was observed by hematoxylin and eosin stain in sections taken from representative histological samples and was further confirmed through immunostaining and quantitation of cellular coverage of the ECM, which demonstrated that 51% of scaffold area was overlaid by epithelial cells at day 9 (Figure 8B, Supplemental Figure 8A, and Supplemental Video 1). A minor limitation is that the cellular coverage quantification methodology is imperfect. Given the whole scaffold matrix area was quantified, including vascular and interstitial spaces, the methodology underestimates alveolar wall coverage. However, in some instances, the quantification overestimates coverage, such as when tissue orientation makes it difficult to discern if one or both sides of the alveolar septa are fully epithelialized. Cell proliferation was observed in the scaffolds and was highest in T2-Max, with 12% Ki-67–positive cells at day 3, and lowest in T1-Base, with 1% Ki-67–positive cells at day 9 (Supplemental Figure 8, B and C). Cell differentiation coincided with the reduction in proliferation, whereby immunostaining revealed the induction of AT1 marker expression, GPRC5A, paired with a gradual loss of AT2 markers, HT2-280 and pSP-C, over time (Figure 8, C and D). By day 9, only 0.8% cells expressed pSP-C, indicating their strong propensity to differentiate in the context of the scaffold (Supplemental Figure 8D). Expression of additional mature AT1 markers, HT1-56, caveolin-1, EMP2, and RAGE, was also detected by immunostaining at the end of culture in T1-Base, which further confirmed differentiation was underway (Supplemental Figure 8, E and F). Quantification of the percentage of RAGE-positive cells in T1-Base revealed that 36% expressed RAGE on average, with variation based on donor (Supplemental Figure 8G). RNAscope analysis further supported these findings, demonstrating an increasing number of *AGER* RNA molecules per cell with time in the scaffold and a higher proportion of *AGER*-positive cells by end of culture compared with RAGE protein expression (76%–99% vs. 36%, Supplemental Figure 8, H and I).

Bulk gene expression by quantitative real-time PCR also revealed a gradual reduction in AT2 marker expression (*SFTPC*, *ABCA3*, *NAPSA*) that aligned with increased AT1 marker expression (*ANKRD1*, *CLIC5*, *GPRC5A*, *PDPN*) (Figure 8E). To further investigate cell identity and cell state in the tissue, single-nucleus RNA-seq (snRNA-seq) was performed and confirmed the trend in differentiation noted by the bulk gene expression analysis. There was a clear, stepwise decrease in AT2 markers, *SFTPC* and *SFTPA1*, and a corresponding stepwise gain in AT1 markers, *AGER* and *CLIC5* (Figure 8F). Applying the Habermann label transfer method used in Figure 3 to our cells in the scaffold revealed that proliferating epithelial cells were the largest population in T2-Max, transitional AT2s were the largest population in T1-Diff and T1-Base, and AT1s were increasing in proportion over time in culture (Supplemental Figure 8J). The transitional AT2 cluster was enriched for some previously defined transitional genes but was most readily characterized by reduced AT2 and increased AT1 marker expression (Supplemental Figure 8, K and L). As with our expansion dataset (Figure 3), there was a negligible population of *KRT5^–^KRT17^+^* aberrant basaloid cells. Integration of our expansion and recellularization sequencing datasets further reinforced the differentiation trajectory observed in the scaffold, as *SFTPC* expression trended lower even in T2-Max compared with P2 and *AGER* expression did not increase until the scaffold T1-Diff phase (Supplemental Figure 8M). Pathway analysis comparing culture time points in the scaffold showed enrichment for cell cycle–related pathways in T2-Max and ECM remodeling pathways in T1-Base in alignment with expected behaviors of proliferating AT2 and differentiating AT1, respectively (Supplemental Figure 8N).

As noted previously, a critical role for AT1s in vivo is the secretion of VEGF (59). ELISA data using conditioned media collected during the recellularization study showed the gradual induction of VEGF-A secretion, recapitulating a function role for AT1 in the scaffold (Figure 8G). Overall, expanded AT2s cultured in the lung scaffold recapitulated their in vivo propensity to restore alveolar coverage through AT1 differentiation, while also retaining a small population of pSP-C–positive progenitors. Supporting a larger proportion of mature AT1s and pSP-C–positive AT2s will likely require further culture medium supplementation and the incorporation of additional cell types, namely the Axin2-positive mesenchymal cells that appear critical to supporting the alveolar epithelial niche (61).

## Discussion

Regenerative medicine is an attractive approach to treating chronic respiratory diseases. However, a major challenge with any lung regenerative medicine approach is obtaining the required number of distal epithelial cells, specifically AT2s (17). Large-scale, bioreactor culture using microcarriers for growth of adherent cells has demonstrated the scalability required to meet yield demands for cell-based therapies (62–65). Our application of bioreactor cultures to primary AT2 expansion leverages this scalability to render lung recellularization feasible. This culture platform is adaptable based on the number of input cells available from isolation, as demonstrated from using different scales, including 250 mL spinner flasks to 10 L bioreactors. The equipment requirement for spinner flasks is minimal and can be found in most labs, requiring only a stir plate and incubator, enabling widespread applicability of this platform. While bioreactor cultures require specialized equipment, they allow precise control over the culture microenvironment, which is advantageous for cell manufacturing and generating consistent yield and quality.

Toward the development of our large-scale expansion method, we sought to decipher the critical cues that maintain AT2s and elucidate the variables driving loss of AT2 phenotype in traditional tissue culture plates. It has been widely demonstrated that stem cells can be influenced by far more than simply the soluble cues generated from nearby cells. They can also sense and respond to the mechanics of their microenvironment, which has been shown to be powerful enough to dictate lineage commitment in mesenchymal stem cells (MSCs) (66–70). The relevance of biophysical cues has also been demonstrated in lung epithelial biology, where mechanical forces were found essential to AT1 differentiation during development and AT1 maintenance during adulthood, while stiffness cues were found to influence the persistence or resolution of transitional states in models of fibrosis (48, 71, 72). Extensive characterization of AT2s has also revealed a remarkable plasticity in this regenerative cell population whose fate is acutely sensitive to the local niche (1, 3–5). Taken together, we hypothesized that maintenance of AT2s would require a deliberate choice in substrate and surface composition, in addition to a carefully designed media composition that targets relevant signaling pathways (3, 4, 22, 34, 73–75).

With microenvironmental responsiveness and biophysical cues in mind, our culture method pairs established mitogenic cues and pathway regulators, which stimulate AT2 proliferation and maintain AT2 phenotype, with a substrate composition and stiffness designed to closely match the matrix and mechanical properties of the alveolar niche. The gelatin microcarriers utilized in our culture platform share a similar chemical composition to collagen, a major component of alveolar ECM, but with altered adhesion receptor recognition sites due to the denaturation process (76). While initially counterintuitive, our mechanistic data revealed that gelatin, rather than collagen, better supported AT2 phenotype maintenance and proliferation. Moreover, we observed limited AT2 spreading on gelatin compared with collagen in our studies. Revisiting the MSC literature revealed restriction of MSC size, and shape is also a potent driver of cell fate (77–80). Combined with the known differences in surface area coverage by AT2s versus AT1s in vivo, we speculated the limited spreading of AT2s on gelatin due to differential adhesive ligand presentation in gelatin compared with collagen aids in maintaining AT2s while preventing AT1 differentiation in our culture platform. This concept of cell spreading as a driver of AT2-AT1 differentiation was recently reported using micropatterned surfaces of different sizes, providing additional support for our hypothesis (48).

Examination of gene and protein expression in expanded AT2s indicated some changes but overall substantial retention of key AT2 characteristics through P4 or P5. Proliferation driven by T2-Max allowed for generation of hundreds of billions of cells from a single donor’s tissue without substantial loss of phenotype or differentiation capacity. Compared with isoAT2s, our expanded AT2s demonstrated limited upregulation of a few genes associated with transitional cell states but did not exhibit gene signatures typical of intermediate populations arising from injury or disease (30, 32, 33). This led us to hypothesize that, rather than inducing an injury repair process, our expansion method engages a regenerative response, likely driven by KGF and EGF in T2-Max, found to stimulate proliferation in AT2s after pneumectomy (45, 81–83). In addition to these mitogenic cues, the inclusion of A-83-01 and DMH-1, inhibitors of TGF-β and BMP pathways, block critical cues that precede AT1 differentiation in vivo (34). Single-cell sequencing provided additional evidence that our expanded cells largely consisted of AT2s, similar to their freshly isolated counterparts but with a larger population of cycling AT2s, as expected from culture in T2-Max media. Reference-based label-transfer cell typing using the Habermann dataset provided further support that our expanded AT2s retained a native-like phenotype with only a small fraction of cells labeled as transitional AT2 (37). This transitional population overlapped with our alveolar cluster 4 and expressed some markers previously reported to arise during homeostatic turnover and lung injury. However, it was identified most clearly by the intermediate expression of both AT2 and AT1 markers, leading us to hypothesize these cells are primed for AT1 differentiation.

In addition to demonstrating that expanded AT2s retain a native-like phenotype, we also evaluated their capacity to undergo AT1 differentiation. Through engagement of the YAP pathway, T1-Diff promoted expression of highly specific AT1 markers. Sustained nuclear localization of aYAP was observed even after removal of the LATS inhibitor, which, combined with the expression or secretion of AT1-specific ligands, junctional markers, and ECM proteins, suggests AT1s can be generated from our expanded AT2s. This capacity was recapitulated in a native-like environment through the recellularization of porcine lung scaffolds, where expanded AT2s from 2 donors demonstrated robust engraftment and differentiation with expression of AT1-specific gene and protein markers. snRNA-seq of tissue sampled across recellularization time points further indicated that expanded AT2s can both proliferate and differentiate in the scaffold. Label transfer from Habermann et al. identified both AT1 and transitional AT2 populations in our dataset, with the latter being the largest fraction in both T1-Diff and T1-Base (37). Given that the transitional state can comprise cells along a differentiation trajectory that simultaneously express markers of both AT2 and AT1, we hypothesize that our transitional population would further mature in the scaffold given more time and with the incorporation of additional cell types to provide more supportive signaling cues than are present in T1-Base alone. This proof-of-concept recellularization demonstrates the utility of the cells produced using our expansion method and provides a foundation for incorporating additional cell types to rebuild vascular and proximal airway structures. Of note, expanded cells from 2 donors have been used to repopulate the distal airspaces of more than 100 left piglet decellularized lung scaffolds each (131 scaffolds for D7V and 120 scaffolds for 9VX). In summary, this AT2 expansion platform presents a solution to a major challenge facing lung regeneration and should enable more widespread use of this critical lung stem cell.

## Methods

### Sex as a biological variable

There were no organ donation exclusion criteria associated with sex assigned at birth. Lung donations were received from both males and females. A majority of the organ donors were male. Sex assigned at birth was not considered as a biological variable.

### Human lung tissue

Human lung tissues declined for transplant were used herein. Acceptance criteria included, but was not limited to, donors between the ages of 13–65 years, with a smoking history of less than 20 pack-years, and without history or current diagnosis of lung disease. A random 3-character unique identifier was assigned to each donor for identification in this paper.

### AT2 isolation

The AT2 isolation protocol used herein is adapted from previously published methods (84). Briefly, the airway was cannulated and lavaged with 4 washes using HBSS (-MgCl_2_/-CaCl_2_) and at least 2 washes HBSS (+MgCl_2_/+CaCl_2_). Following lavage, elastase (20 U/mL) and type IV collagenase (300 U/mL) were instilled into the airway and incubated for approximately 45 minutes. Tissue was dissociated by hand and filtered through mesh to remove undigested pieces of tissue. Fetal bovine serum was then added to the cell suspension, which was centrifuged for 8 minutes at 300*g*. Cell separation was performed using a depletion strategy, whereby cells were labeled with CD45, CD90, and CD271 microbeads and the non-AT2s were magnetically separated using CliniMACS Plus and/or a MultiMACS instrument(s). Cells were cryopreserved in CryoStor CS10 prior to expansion.

### Cell culture media

For preparation of AT2 expansion culture medium, T2-Max, basal DMEM/F12 medium was supplemented with 5% FBS, 2 �M A-83-01, 2 �M CHIR99021, 1 �M DMH-1, 10 �M Y-27632, 50–100 ng/mL FGF-7, 50 ng/mL EGF, and 100 �g/mL Primocin. For AT1 differentiation culture medium, T1-Diff, basal DMEM/F12 medium was supplemented with 5% FBS, 10 �M Lats-IN-1, and 100 �g/mL Primocin. AT1 maturation medium, T1-Base, comprises DMEM/F12 medium supplemented with 5% FBS and 100 �g/mL Primocin.

### Cell culture

#### Microcarrier culture.

Gelatin microcarriers (Percell Biolytica) were hydrated in Dulbecco’s PBS (DPBS) for 3 hours at room temperature and then autoclaved using a liquids cycle. DPBS was exchanged for T2-Max medium and equilibrated in an incubator (37°C, 5% CO_2_) for 2 hours on a magnetic stirrer. AT2s were seeded at 10,000 cells/cm^2^, with intermittent agitation for 19 hours. For P0 cultures (defined as the first time cells were seeded on microcarriers), the number of cells seeded was adjusted for cell banks with less than 70% of cells stained with HT2-280 to maintain 10,000 HT2-280^+^ cells/cm^2^. The cell-laden microcarrier suspension was continuously agitated for the remainder of the culture with a partial media exchange every 2–3 days. In-process cell counts were obtained by removing 3 mL of the culture. Microcarriers were allowed to settle, and supernatant was transferred into a fresh tube for analysis on a Nova Biomedical NovaFLEX2. Lysis-1 buffer (Chemometec, catalog 910-0010) was added to microcarriers, and cell counts were determined by quantification of nuclei using a Chemometec Nucleocounter NC-202 Cell Counting and Viability protocol. Cells were harvested from microcarriers using 0.25% trypsin or TrypLE with agitation for 15–20 minutes. For serial passaging, the passage number was increased upon seeding cells into a new culture vessel (Figure 1A). When bioreactors were seeded from the same cell stock, cultured in parallel, harvested together, and subsequently pooled, they were grouped into what was defined as a cell lot. Given the large size of these cell banks, for the later passages, multiple cell lots were often generated from a single donor. We utilized attached cells counts (20–24 hours after inoculation) to determine the starting cell number in our “Culture PDL” calculation, and the final cell number was determined, prior to harvest, on the final culture day.

#### AT1 differentiation.

Glass-bottom chamber slides, standard polystyrene plates, or 6.5 mm polyethylene terephthalate Transwell inserts were coated with 20 �g/mL rat tail collagen-I for at least 1 hour at room temperature and inserts were rinsed before seeding. Cryopreserved isoAT2s or P2 AT2s were thawed and cryomedia was slowly diluted with T2-Max and seeded at a density of 2 × 10^4^ cells/cm^2^ in T2-Max. After 2 days in culture, medium was switched to T1-Diff for 3 days, followed by culture in T1-Base for 3 more days, with a media replacement on day 7. For AT1 gene expression analysis, samples were normalized to donor-specific AT2 controls run for each experiment.

### Immunofluorescence-based assays

Methods were adapted based on sample type and imaging modality to be used, inclusive of flow cytometry, wide-field and confocal microscopy (inclusive of a high-content imaging confocal instrument), and an imaging cytometer. For the most part, standard IHC/IF techniques were followed. Detailed descriptions for each sample type are included in the Supplemental Methods.

### Recellularization

Porcine lung scaffolds were produced using a previously published method (85). Briefly, lungs from 6- to 9-week-old Yorkshire/Duroc/Landrace crossed pigs were surgically procured and reduced to intact left lung grafts, which were then decellularized using an automated process by performing successive airway insufflation and expulsion cycles with 1X PBS containing antibiotics/antimycotics, 0.1% Triton X-100, 2% sodium deoxycholate, and 1 M NaCl. Deionized water washes were performed between each detergent and salt solution treatment to clear lysed cellular components and residual solutes. Lung scaffolds were stored for up to 3 weeks at 4ºC in PBS containing antibiotics/antimycotics.

Recellularization of lung scaffolds was accomplished using a custom bioreactor system that facilitated scaffold perfusion and cell seeding under flow and/or pressure control in a proprietary, semiautomated process. Briefly, 6 lung scaffolds were cannulated via the bronchus, pulmonary artery, and left atrium (containing pulmonary veins) and installed into a bioreactor chamber that interfaced aseptically with the control hardware such that the system provided fluid flow to and from each of the major cannulated lung compartments. P2 AT2s from 2 donors were seeded twice with 1e9 cells per seed, with 24 hours between each seeding, into the airway of the lung scaffolds in T2-Max medium. After a 2-hour attachment period with gentle fluid instillation (10 mL/minute to 100 mL every 10 minutes), airway oscillatory feeding (insufflation and fluid expulsion cycles) began at a rate of 130 mL per insufflation and 15 cycles per hour. After 3 days of culture under these conditions, the medium was changed to T1-Diff, and culture was continued for 3 more days. On the sixth day of culture, the medium was changed to T1-Base, and culture was continued for an additional 3 days, for a total of 9 days in culture, day 0 being the first. All media formulations are the same as listed above in the *Cell culture media* section, but Primocin was replaced with a combination of 2X antibiotic-antimycotic, 20 �g/mL gentamicin sulfate, and 0.5 �g/mL amphotericin B for the recellularization cultures. Two engineered lungs were assessed at each culture takedown time point (days 3, 6, and 9).

### Supplemental Methods

Methods not covered herein can be found in the supplemental material.

### Declaration of AI-assisted technologies in the writing process

Authors used ChatGPT4.0 to edit specific sentences, in the Results only, for grammar and conciseness on February 22, 2024, and March 21, 2024. No sentences containing data, analysis, or interpretation were generated or edited with ChatGPT. After using this tool, authors reviewed and edited the content and assume full responsibility for the content of this article.

### Statistics

Statistical analysis was performed using GraphPad Prism. Statistical tests include mixed effects analysis with Tukey’s multiple comparisons test, unpaired 2-tailed *t* test, repeated measures 1-way ANOVA with Tukey’s multiple comparisons test, generalized linear model quasi-likelihood f test, 1-way ANOVA with Dunnett’s post hoc test, and 1-way ANOVA with Tukey’s multiple comparisons test. A *P* value of less than 0.05 was considered significant.

Cell size analysis was conducted using Phenix Harmony software for 3 AT2 donors over 4 time points. The analyzed images were reviewed and in rare instances where the software quantified acellular regions of interest, we excluded them from analysis. To equally weigh the data from each donor, the donor with the lowest number of quantified cells per time point was identified, and all analyzed cells were included for that donor. For the other 2 donors, an equivalent number of cells were then selected in numerical order based on the regions of interest number assigned by software.

### Study approval

Human lung tissue was procured through organ procurement organizations operating under the certifications and standards set forth by the appropriate governing bodies.

### Data availability

Data can be found in the Supporting Data Values file. RNA-sequencing data were deposited in NCBI GEO (accession ID GSE306194 and GSE306714).

## Author contributions

LKR, DFR, RJO, RSV, LCR, SSH, and THP conceptualized the research. RJO, LKR, RSV, SSH, DFR, DWM, RWB, AKS, TRZ, LCR, and BAF designed the experiments. LKR, RJO, RSV, DFR, ARD, JCW, and DWM conducted the experiments. LKR, RSV, RJO, DPK, AKS, JCW, and LCR performed data analysis. LCR, LKR, RJO, and RSV wrote the manuscript. LCR, THP, BAF, TRZ, SSH, and RWB edited the manuscript. LCR, SSH, RWB, and THP supervised the work.

## Conflict of interest

This work was funded by United Therapeutics. All authors are or were employed by, receive a salary from, and own equity in United Therapeutics. TRZ’s spouse is employed by and earns a salary from UNC Health. DPK’s spouse is employed by and earns a salary from Powered Research. See Supplemental Data for information on patents and patent applications related to this work.

## Funding support

Given this work is the result of NIH funding, in part, it is subject to the NIH Public Access Policy. Through acceptance of this federal funding, the NIH has been given a right to make the work publicly available in PubMed Central.

- P30 CA016086 Cancer Center Core Support Grant to the UNC Lineberger Comprehensive Cancer Center to Microscopy Services Laboratory, Department of Pathology and Laboratory Medicine.
- United Therapeutics.

## Supplementary Material

Supplemental data

Unedited blot and gel images

Supplemental table 1

Supplemental table 2

Supplemental video 1

Supporting data values

## Version 1 06/01/2026

Electronic publication
