# Supplementary material for: Leveraging factors that control alveolar epithelial cell fate enables large-scale expansion for lung tissue engineering
Source: J Clin Invest. 2026 Jun 1;136(11):e188701. doi: 10.1172/JCI188701 (PMC13221229; doi:10.1172/JCI188701)
Supplement: Supplemental data [file jci-136-188701-s279.pdf]

Supplemental Figures

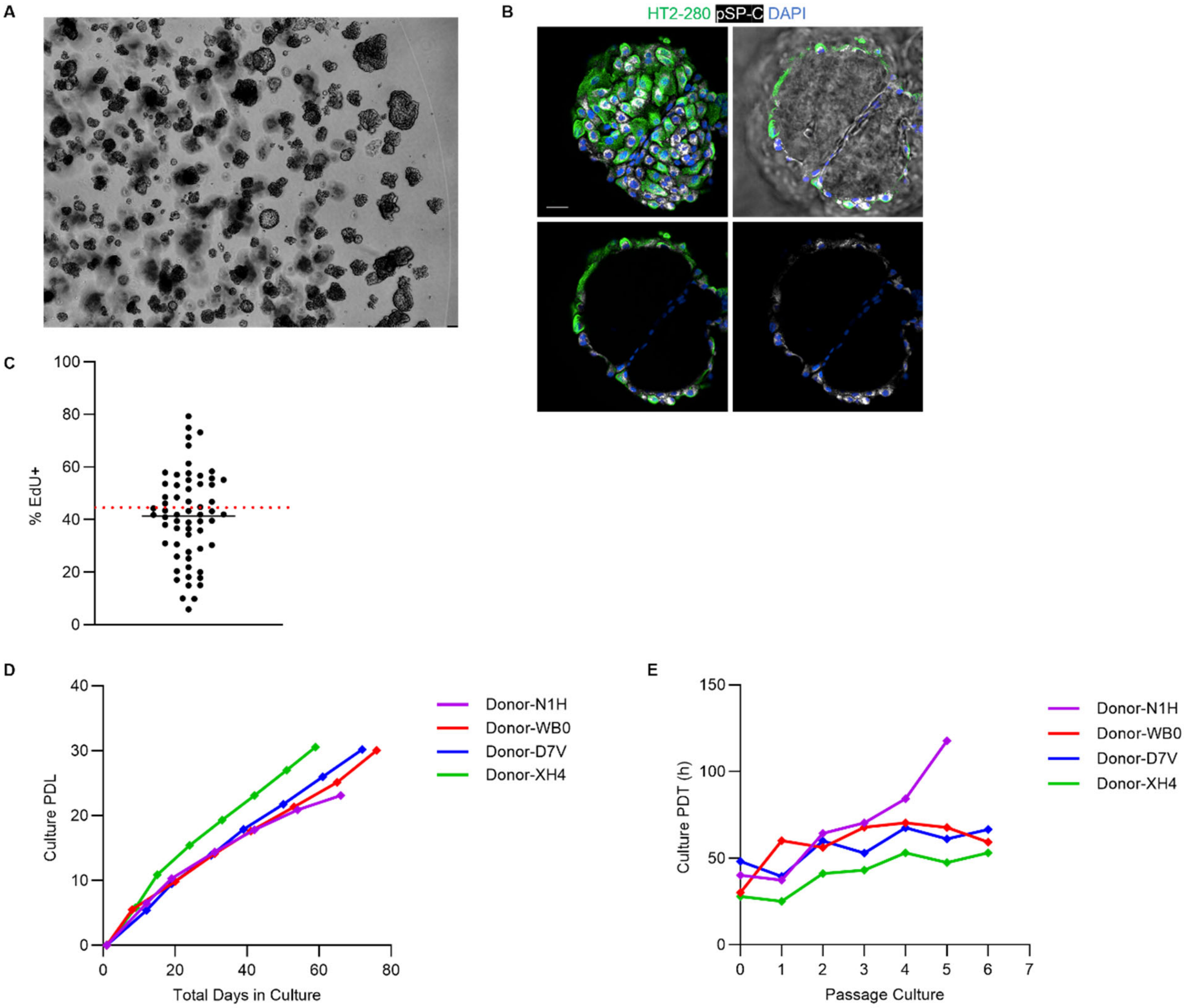

F

Donor	Initial	Passage 0	Passage 1	Passage 2	Passage 3	Passage 4	Passage 5	Passage 6
**N1H**	NT	Normal; nonclonal finding	Normal	Normal	Normal	NT	1) Normal 2) Abnormal (trisomy 18)	NT
**WB0**	NT	Normal; nonclonal finding	Normal; nonclonal finding	Normal	Normal; nonclonal finding	Normal	Normal; nonclonal finding	Normal; nonclonal findings
**XH4**	NT	Normal	1) Normal 2) Normal; nonclonal finding	Normal; nonclonal findings	Normal	Normal	Normal; nonclonal finding	Normal
**D7V**	NT	NT	Normal; nonclonal finding	Abnormal (trisomy 12); nonclonal findings	Normal; nonclonal findings	Normal; nonclonal finding	Normal	Normal; nonclonal finding
**V5M**	Normal	Normal; nonclonal finding	Normal	NT	NT	NT	NT	NT
**KP9**	Normal; nonclonal finding	Normal; nonclonal finding	Normal	Normal	NT	NT	NT	NT
**TN3**	NT	Normal	NT	Normal	NT	NT	NT	NT

Supplemental Figure 1. Characterization of AT2 proliferative capacity. Related to Fig. 1.

- **(A)** Phase image of AT2 cells cultured in Matrigel in T2-Max for 9 days. Scale bar, 100 µm.
- **(B)** A second example, with cells from a different donor, of immunostaining for AT2-specific proteins on AT2s on microcarriers on day 11 of passage 3: HT2-280 (green), pSP-C (white), DAPI (blue). Upper left: maximum projection; upper right: microcarrier cross-section, brightfield merge; lower left: microcarrier cross-section, without brightfield merge; lower right: microcarrier cross section with pSP-C and DAPI only. Scale bar, 50 µm.
- **(C)** Graph of EdU data from isoAT2 across cell lots (in some cases, multiple cell lots are generated from a single donor). Dotted red line indicates pass/fail criteria.
- **(D)** Graph displaying the cumulative culture PDL over 5-6 successive cultures for AT2 cells isolated from 4 donors.
- **(E)** Graph displaying the culture PDT over 5-6 successive cultures for AT2 cells isolated from 4 donors.
- **(F)** Table of karyotyping results from multiple passages across 7 donors. NT, not tested.

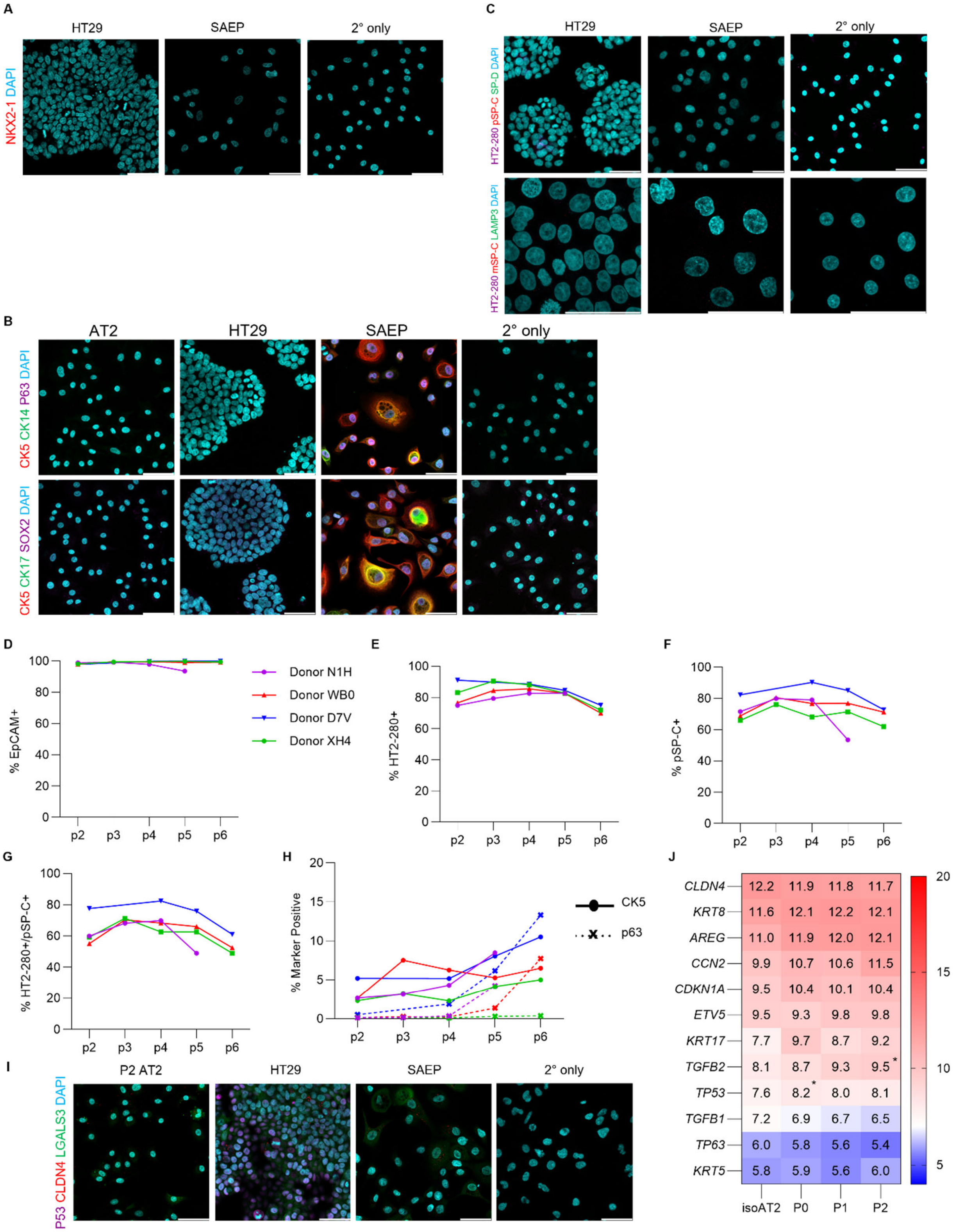
A
HT29
SAEP
2° only
NKX2-1 DAPI
C
HT29
SAEP
2° only
HT2-280 pSP-C SP-D DAPI
HT2-280 mSP-C LAMP3 DAPI
B
AT2
HT29
SAEP
2° only
CK5 CK14 P63 DAPI
CK5 CK17 SOX2 DAPI
D
% EpCAM+
Donor N1H
Donor WB0
Donor D7V
Donor XH4
E
% HT2-280+
F
% pSP-C+
G
% HT2-280+/pSP-C+
H
% Marker Positive
CK5
p63
p2
p3
p4
p5
p6
J
CLDN4 12.2 11.9 11.8 11.7
KRT8 11.6 12.1 12.2 12.1
AREG 11.0 11.9 12.0 12.1
CCN2 9.9 10.7 10.6 11.5
CDKN1A 9.5 10.4 10.1 10.4
ETV5 9.5 9.3 9.8 9.8
KRT17 7.7 9.7 8.7 9.2
TGFB2 8.1 8.7 9.3 9.5*
TP53 7.6 8.2* 8.0 8.1
TGFB1 7.2 6.9 6.7 6.5
TP63 6.0 5.8 5.6 5.4
KRT5 5.8 5.9 5.6 6.0
isoAT2
P0
P1
P2
20
15
10
5
I
P2 AT2
HT29
SAEP
2° only
P53 CLDN4 LGALS3 DAPI

Supplemental Figure 2. AT2 Phenotype Over Continued Expansion and Analysis of Transitional Populations. Related to Fig. 2.

- **(A)** Immunostaining for NKX2-1 (red) in HT-29 enterocytes and small airway epithelial cells (SAEP), with secondary only control. Scale bar, 50 µm.
- **(B)** Immunostaining for CK14 (green), CK5 (red), P63 (magenta) in the top panel and CK17 (green), CK5 (red), SOX2 (magenta) in the bottom panel in P2 AT2s, HT-29 and SAEP with secondary only control. Scale bar, 50 µm.
- **(C)** Immunostaining for SP-D (green), pSP-C (red), HT2-280 (magenta) in the top panel and LAMP3 (green), mSP-C (red), HT2-280 (magenta) in the bottom panel in HT-29 and SAEP, with secondary only control. Scale bar, 50 µm (top panel) and 25 µm (bottom panel).
- **(D)** Flow cytometry analysis of the percent of EpCAM-positive cells from passage 2 through passage 6 AT2s (n=3-4 donors at each passage).
- **(E)** Quantitation of the percent of HT2-280-positive cells over passage by immunostaining (n=3-4 donors at each passage). P2 data from Figure 2 was used herein for E-G.
- **(F)** Quantitation of the percent of pSP-C-positive cells over passage by immunostaining (n=3-4 donors at each passage).
- **(G)** Quantitation of the percent of HT2-280/pSP-C dual positive cells over passage by immunostaining (n=3-4 donors at each passage)
- **(H)** Quantitation of the percent of CK5-positive and p63-positive cells plotted individually over passage by immunostaining (n=3-4 donors at each passage; CK5: closed circle on solid line; P63: X on dashed line).
- **(I)** Immunostaining for LGALS3 (green), Claudin-4 (CLDN4, red), P53 (magenta) in P2 AT2s, HT-29, and SAEP with secondary only control. Scale bar, 50 µm.
- **(J)** Bulk quantitation of mRNA transcripts using nCounter technology in isoAT2s and expanded AT2s (n=6 donors per passage; mean of log2 normalized counts; repeated measures one-way ANOVA, alpha 0.05, Tukey's multiple comparisons test, *$p<0.05$ compared to isoAT2). DAPI (cyan) in A, B, C, and I.

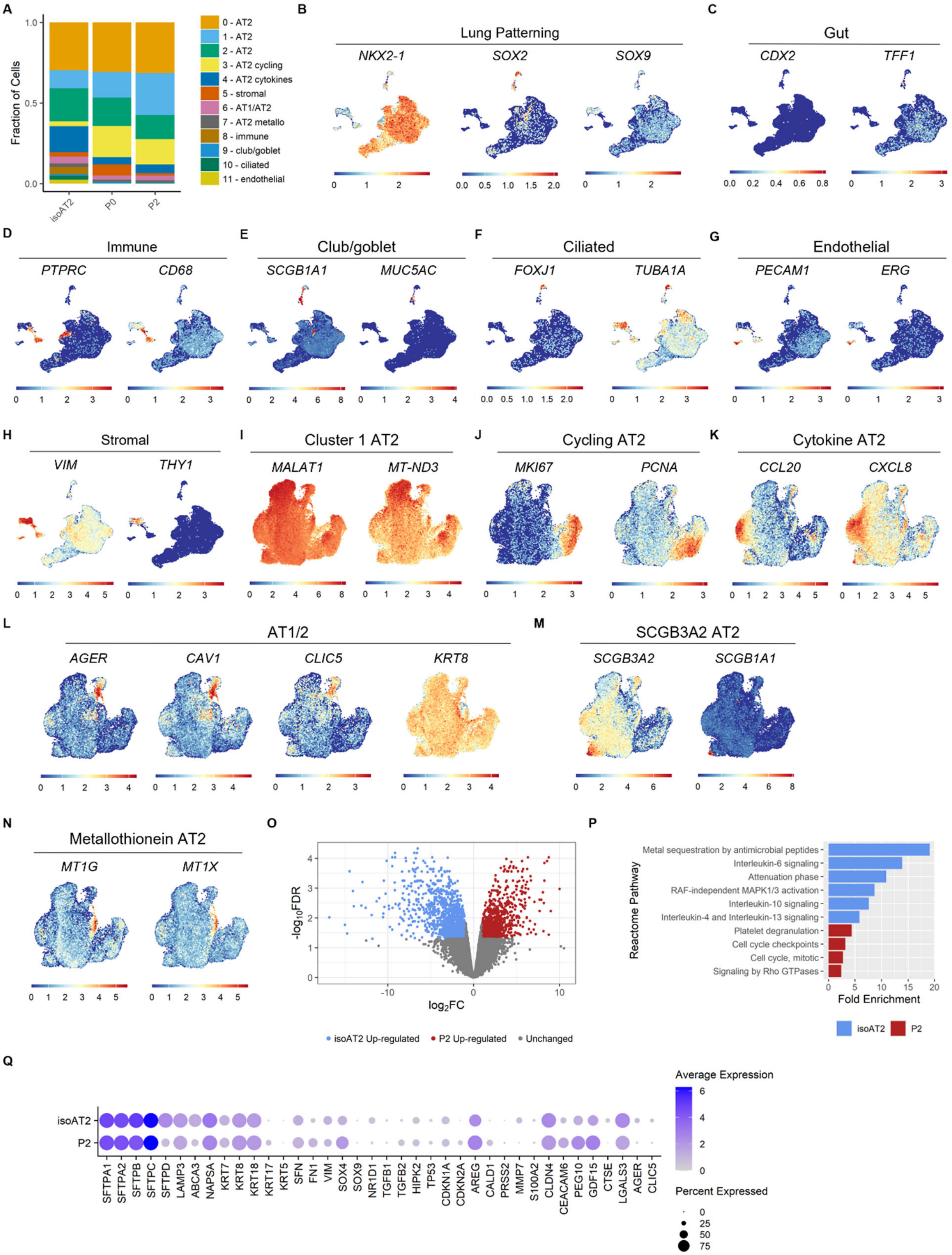

A
Fraction of Cells
isoAT2
P0
P2
0 - AT2
1 - AT2
2 - AT2
3 - AT2 cycling
4 - AT2 cytokines
5 - stromal
6 - AT1/AT2
7 - AT2 metallo
8 - immune
9 - club/goblet
10 - ciliated
11 - endothelial
B
Lung Patterning
NKX2-1
SOX2
SOX9
C
Gut
CDX2
TFF1
D
Immune
PTPRC
CD68
E
Club/goblet
SCGB1A1
MUC5AC
F
Ciliated
FOXJ1
TUBA1A
G
Endothelial
PECAM1
ERG
H
Stromal
VIM
THY1
I
Cluster 1 AT2
MALAT1
MT-ND3
J
Cycling AT2
MKI67
PCNA
K
Cytokine AT2
CCL20
CXCL8
L
AT1/2
AGER
CAV1
CLIC5
KRT8
M
SCGB3A2 AT2
SCGB3A2
SCGB1A1
N
Metallothionein AT2
MT1G
MT1X
O
-log10FDR
log2FC
isoAT2 Up-regulated
P2 Up-regulated
Unchanged
P
Reactome Pathway
Metal sequestration by antimicrobial peptides
Interleukin-6 signaling
Attenuation phase
RAF-independent MAPK1/3 activation
Interleukin-10 signaling
Interleukin-4 and Interleukin-13 signaling
Platelet degranulation
Cell cycle checkpoints
Cell cycle, mitotic
Signaling by Rho GTPases
Fold Enrichment
isoAT2
P2
Q
isoAT2
P2
SFTPA1 SFTPA2 SFTPB SFTPC SFTPD LAMP3 ABCA3 NAPSA KRT7 KRT8 KRT18 KRT17 KRT5 SFN FN1 VIM SOX4 SOX9 NR1D1 TGFB1 TGFB2 HIPK2 TP53 CDKN1A CDKN2A AREG CALD1 PRSS2 MMP7 S100A2 CLDN4 CEACAM6 PEG10 GDF15 CTSE LGALS3 AGER CLIC5
Average Expression
Percent Expressed

Supplemental Figure 3. Additional Single-cell RNA Sequencing Analyses of the Expanded AT2s. Related to Fig. 3.

(A) Proportion plot showing the 12 identified clusters, comparing isoAT2 to P0 and P2.

(B-H) UMAP plots showing lung patterning markers *NKX2-1, SOX2, SOX9* (B), gut markers *CDX2, TFF1* (C), (C), immune markers *PTPRC, CD68* (D), club/goblet markers *SCGB1A1, MUC5AC* (E), ciliated markers *FOXJ1, TUBA1A* (F), endothelial markers *PECAM, ERG* (G), stromal markers *VIM, THY1* (H).

(I-O) UMAP plots showing transcript expression patterns for each alveolar cluster: cluster 1 AT2 *MALAT1, MT-ND3* (I), cluster 2 cycling AT2 *MKI67, PCNA* (J), cluster 3 cytokine AT2 *CCL20, CXCL8* (K), cluster 4 AT1/2 *AGER, CAV1, CLIC5, KRT8,* (L), cluster 5 SCGB3A2+ AT2 *SCGB3A2, SCGB1A1* (M), and cluster 6 metalloproteinase AT2 *MT1G, MT1X* (N).

(O) Volcano plot showing differentially upregulated genes for isoAT2 vs P2 AT2.

(P) Results of a Panther over-representation test against the Reactome pathway database using the differentially expressed genes in O.

(Q) Dot plot of selected transitional cell markers comparing isoAT2 to P2 AT2.

All UMAP plots are expression-ordered.

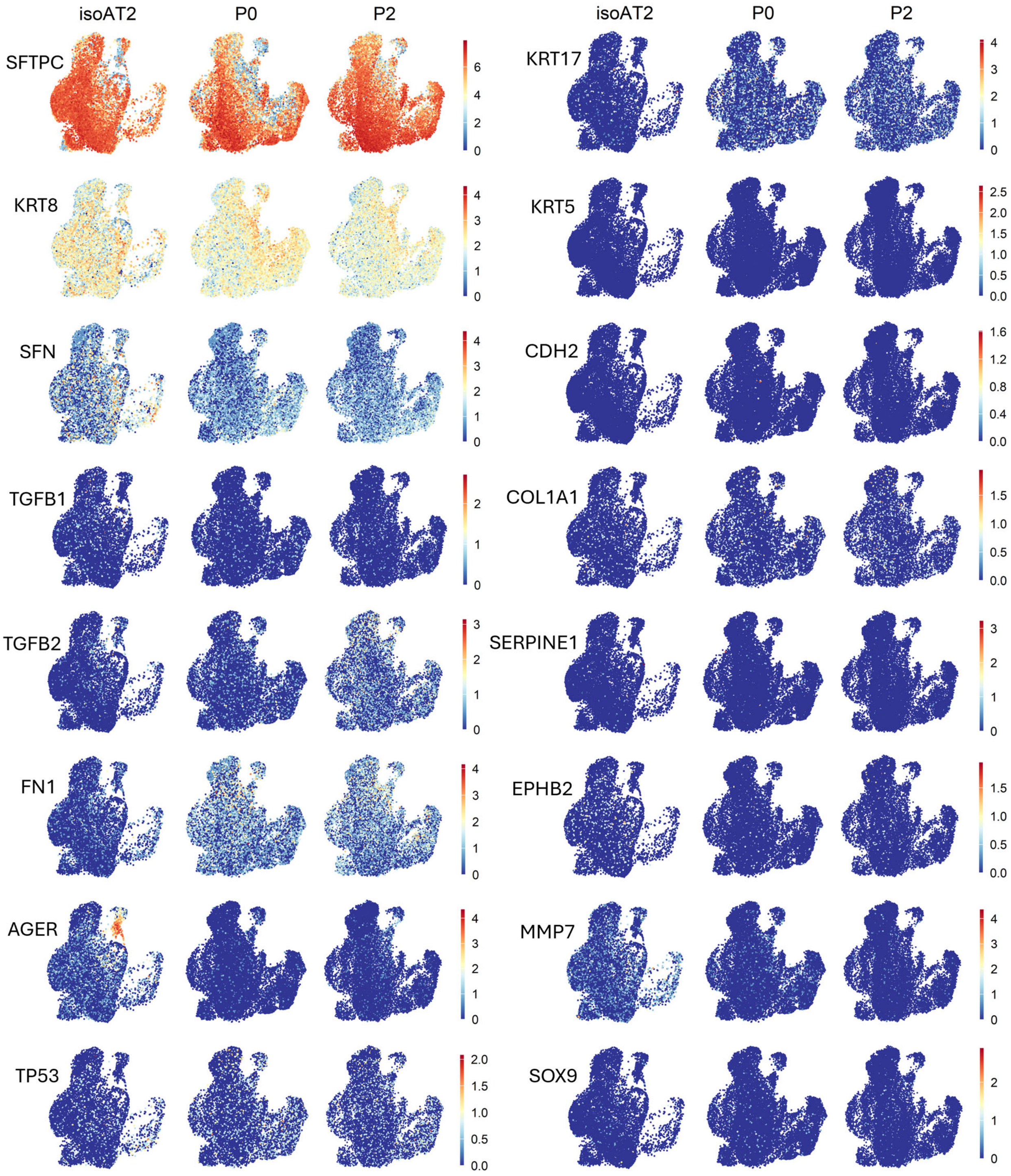

isoAT2
P0
P2
isoAT2
P0
P2
SFTPC
KRT8
SFN
TGFB1
TGFB2
FN1
AGER
TP53
KRT17
KRT5
CDH2
COL1A1
SERPINE1
EPHB2
MMP7
SOX9

Supplemental Figure 4. Additional Single-cell RNA Sequencing Analyses of the Expanded AT2s. Related to Fig. 3.

UMAP plots alveolar clusters only, showing AT2, AT1, transitional, and aberrant basaloid genes.

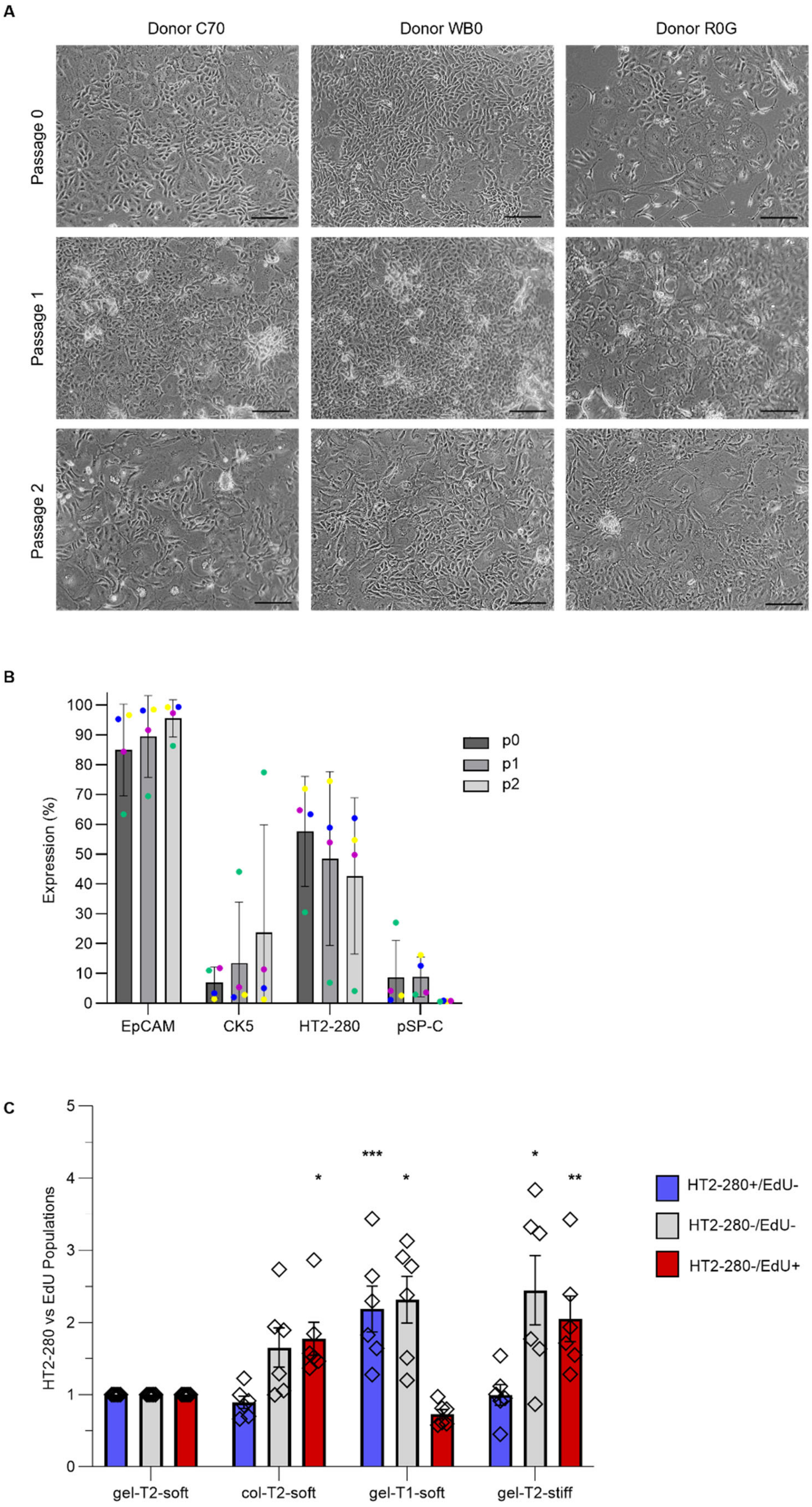
A
Donor C70
Donor WB0
Donor R0G
Donor VRL
Passage 0
Passage 1
Passage 2
B
Expression (%)
100
90
80
70
60
50
40
30
20
10
0
EpCAM
CK5
HT2-280
pSP-C
p0
p1
p2
C
HT2-280 vs EdU Populations
5
4
3
2
1
0
gel-T2-soft
col-T2-soft
gel-T1-soft
gel-T2-stiff
***
**
*
HT2-280+/EdU-
HT2-280-/EdU-
HT2-280-/EdU+

Supplemental Figure 5. Standard 2D expansion does not maintain an AT2 population. Related to Fig. 4.

- **(A)** Brightfield images prior to harvest for AT2s isolated from 4 donors and seeded onto collagen 1-coated flasks in T2-Max and grown for 3 passages. Scale bar, 200 µm.
- **(B)** Flow cytometry analysis of EpCAM, Ck5, HT2-280, and pSP-C expression from cells grown in panel A following harvest from flasks, n=4 donors (donor-C70: yellow, donor R0G: green, donor WB0: blue, donor VRL: red). C70, WB0, and R0G expanded concurrently, VRL expanded separately. Data represents mean ± SD.
- **(C)** HT2-280 and EdU scatter plot populations from 2D mechanism study. The percentage of cells in each quadrant was normalized by ratio to the control condition (gel-2M-soft). Mean ± SEM of six donors across two independent experiments. One-way ANOVA with Dunnett's post-hoc test; *$p<0.05$,**$p<0.01$,***$p<0.001$.

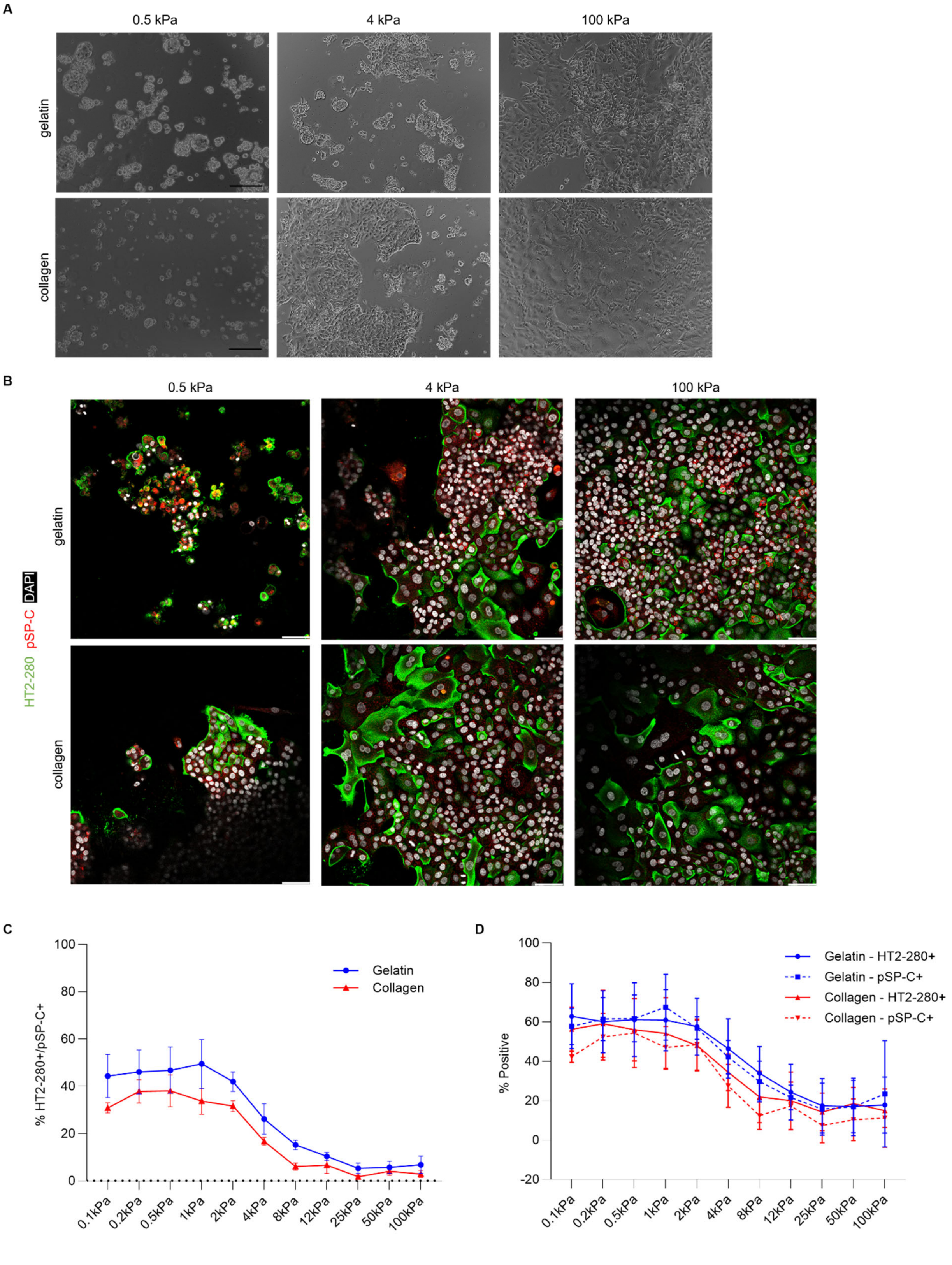
A
0.5 kPa
4 kPa
100 kPa
gelatin
collagen
B
0.5 kPa
4 kPa
100 kPa
gelatin
collagen
HT2-280 pSP-C DAPI
C
% HT2-280+/pSP-C+
Gelatin
Collagen
0.1kPa
0.2kPa
0.5kPa
1kPa
2kPa
4kPa
8kPa
12kPa
25kPa
50kPa
100kPa
D
% Positive
Gelatin - HT2-280+
Gelatin - pSP-C+
Collagen - HT2-280+
Collagen - pSP-C+
0.1kPa
0.2kPa
0.5kPa
1kPa
2kPa
4kPa
8kPa
12kPa
25kPa
50kPa
100kPa

Supplemental Figure 6. Stiffness is a driver of AT2 phenotype changes. Related to Fig. 4.

- **(A)** Brightfield images of AT2 cells grown on representative stiffnesses on gelatin- or collagen-coated substrates. Scale bar, 200 µm.
- **(B)** Representative images of HT2-280 (green), pSP-C (red), and DAPI (white) from stiffness array plates. Scale bar, 50 µm.
- **(C)** Percent of pSP-C/HT2-280 dual positive cells from immunostained stiffness array plates (mean ±SEM of n=3 donors per stiffness, substrate; 2 donor banks were cultured concurrently, with the 3rd cultured independently).
- **(D)** Percent of pSP-C-positive and HT2-280-positive cells from immunostained stiffness array plates coated with gelatin or collagen (mean ±SEM of n=3 donors per stiffness, substrate; 2 donor banks were cultured concurrently, with the 3rd cultured independently).

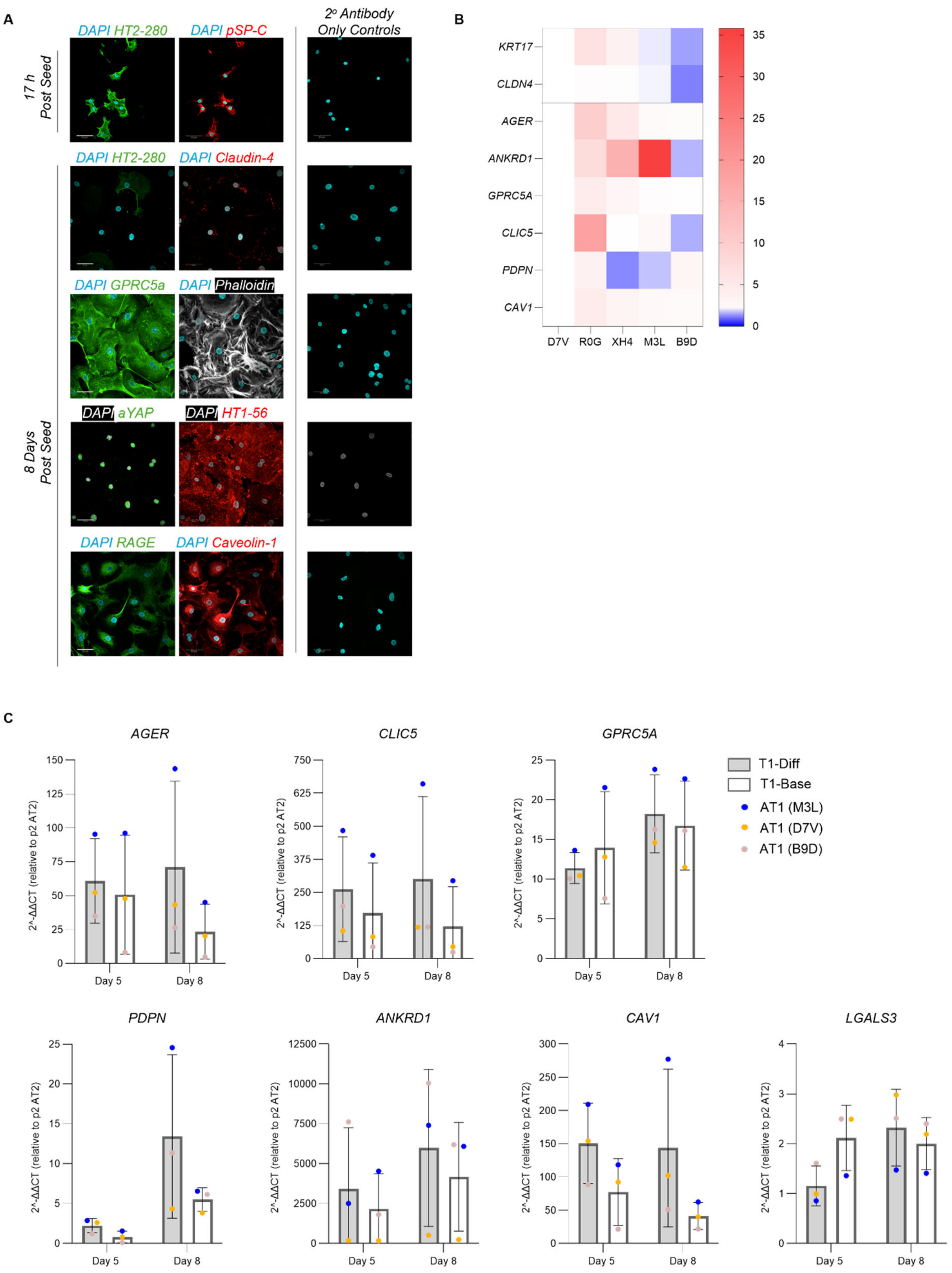
A
DAPI HT2-280
DAPI pSP-C
2° Antibody Only Controls
17 h Post Seed
DAPI HT2-280
DAPI Claudin-4
DAPI GPRC5a
DAPI Phalloidin
DAPI aYAP
DAPI HT1-56
DAPI RAGE
DAPI Caveolin-1
8 Days Post Seed
B
KRT17
CLDN4
AGER
ANKRD1
GPRC5A
CLIC5
PDPN
CAV1
D7V
R0G
XH4
M3L
B9D
35
30
25
20
15
10
5
0
C
AGER
CLIC5
GPRC5A
PDPN
ANKRD1
CAV1
LGALS3
2^-ΔΔCT (relative to p2 AT2)
Day 5
Day 8
T1-Diff
T1-Base
AT1 (M3L)
AT1 (D7V)
AT1 (B9D)

Supplemental Figure 7. Baseline AT1 Gene Expression and AT1 Medium Comparison. Related to Fig. 5.

(A) Images from Figure 5B presented again but as single color immunostaining images in order to clarify the staining pattern of each marker. Secondary antibody only control images are also included. Scale bars, 50 μM.

(B) Gene expression quantification of passage 2 expanded AT2s from 5 donors prior to differentiation in T1-Diff, relative to passage 2 AT2 cells from donor D7V (2^-ΔΔCT).

(C) Gene expression quantification of passage 2 expanded AT2s (n= 3 donors) cultured in T1-Diff and T1-Base on days 5 and 8, relative to the passage 2 cells before seeding (2^-ΔΔCT), showing AT1 markers, *AGER*, *ANKRD1*, *GPRC5A*, *CLIC5*, *PDPN*, and *CAV1*, and the transitional marker, *LGALS3*. T1-Diff data from Figure 5 for donors M3L and B9D was used herein for comparison to the T1-Base condition.

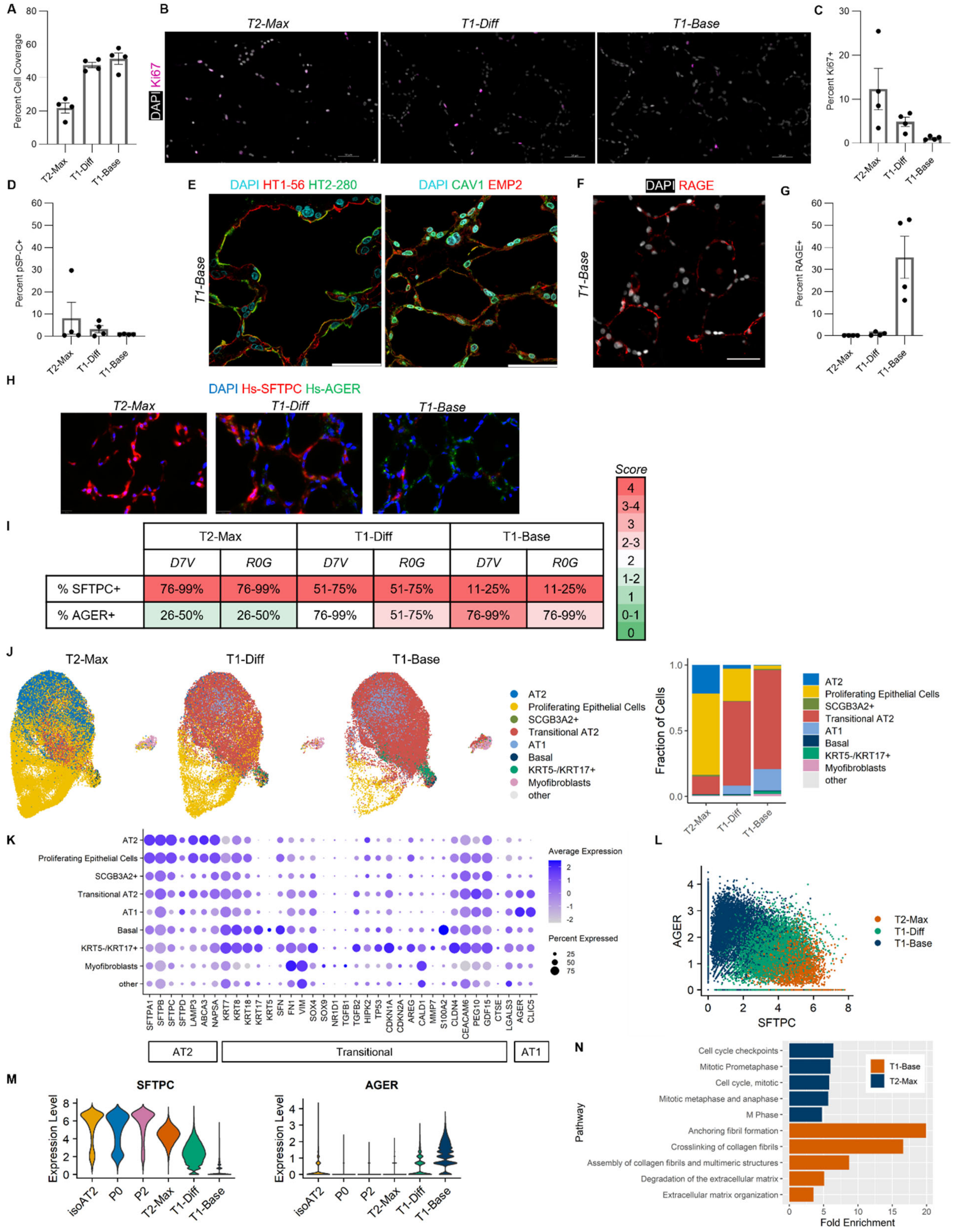
A
Percent Cell Coverage
T2-Max
T1-Diff
T1-Base
B
DAPI Ki67
C
Percent Ki67+
D
Percent pSP-C+
E
DAPI HT1-56 HT2-280
DAPI CAV1 EMP2
F
DAPI RAGE
G
Percent RAGE+
H
DAPI Hs-SFTPC Hs-AGER
I
Score
4
3-4
3
2-3
2
1-2
1
0-1
0
T2-Max | T1-Diff | T1-Base
D7V | R0G | D7V | R0G | D7V | R0G
% SFTPC+ | 76-99% | 76-99% | 51-75% | 51-75% | 11-25% | 11-25%
% AGER+ | 26-50% | 26-50% | 76-99% | 51-75% | 76-99% | 76-99%
J
AT2
Proliferating Epithelial Cells
SCGB3A2+
Transitional AT2
AT1
Basal
KRT5-/KRT17+
Myofibroblasts
other
Fraction of Cells
K
Average Expression
Percent Expressed
SFTPA1 SFTPB SFTPC SFTPD LAMP3 ABCA3 NAPSA KRT7 KRT8 KRT18 KRT17 KRT5 SFN FN1 VIM SOX4 SOX9 NR1D1 TGFB1 TGFB2 HIPK2 TP53 CDKN1A CDKN2A AREG CALD1 MMP7 S100A2 CLDN4 CEACAM6 PEG10 GDF15 CTSE LGALS3 AGER CLIC5
AT2
Transitional
AT1
L
AGER
SFTPC
M
SFTPC
AGER
Expression Level
isoAT2
P0
P2
N
Cell cycle checkpoints
Mitotic Prometaphase
Cell cycle, mitotic
Mitotic metaphase and anaphase
M Phase
Anchoring fibril formation
Crosslinking of collagen fibrils
Assembly of collagen fibrils and multimeric structures
Degradation of the extracellular matrix
Extracellular matrix organization
Pathway
Fold Enrichment

Supplemental Figure 8. Additional Characterization of AT2s and AT1s in Receullarized Scaffolds. Related to Fig. 7.

- **(A)** Estimation of cell coverage in recellularization experiments by quantifying the amount of CK8 cell area overlapping COL4 scaffold area as a percentage of total scaffold area (n=4 samples per time point; mean ± SEM).
- **(B)** Representative images of Ki-67 (magenta) and DAPI (white) immunostaining in recellularization experiments. Scale bars, 50 µm.
- **(C)** Quantitation of percent of Ki-67-positive cells (n=4 samples per time point; mean ± SEM).
- **(D)** Quantitation of pSP-C immunostaining over recellularization time course (n=4 samples per time point; mean ± SEM).
- **(E)** Additional AT1 immunostaining of DAPI (cyan, all), HT1-56 (red, left), HT2-280 (green, left) and CAV1 (green, right) and EMP2 (red, right) in T1-Base media.
- **(F)** Immunostaining of RAGE (red) and DAPI (white) in T1-Base.
- **(G)** Quantitation of percent of RAGE-positive cells over recellularization time course (n=4 samples per time point; mean ± SEM).
- **(H)** RNAscope of *SFTPC* (red) and *AGER* (green) over recellularization time course.
- **(I)** Semi-quantitation of RNAscope samples reporting a range of percent of positive cells and a score, indicated by color (4: >15 dots/cell and/or >10% dots are in clusters; 0: no staining or <1 dot/10 cells).
- **(J)** UMAP and proportion plot from single nucleus RNA-seq (snRNA-seq) data showing cell types identified by a label transfer method using the Habermann, et al. dataset (1).
- **(K)** SnRNA-seq data showing a dot plot of transitional cell markers by cluster.
- **(L)** SnRNA-seq data showing a scatter plot of *SFTPC* vs *AGER* expression of cells only from the Transitional AT2 cluster over the recellularization time course, T2-Max (orange), T1-Diff (green), and T1-Base (blue).
- **(M)** RNA-seq data shown as violin plots of *SFTPC and AGER* from integration of the expansion and recellularization datasets.
- **(N)** Results of a Panther Classification System over-representation test against the Reactome pathway database to show the top enriched pathways comparing cells in T2-Max vs T1-Base from the snRNA-seq dataset.

Supplementary Video 1. Related to Figure 7. Video generated using QuPath showing increasing magnification and panning around on a scan of a megaslide of the caudal lobe of R0G in T1-Base, showing markers Cytokeratin-8 (Magenta), HT2-280 (green), GPRC5a (red), and DAPI (white).

Supplementary Table 1. Related to Figure 3. Marker genes identified for each alveolar cluster.

Supplementary Table 2. Related to Figure 3. Results of differential gene expression analysis for passage 2 vs isoAT2 cells.

Patent Information

Two patent applications related to this work have been filed (US 17/003,431 and US 17/894,709; associated related applications: 22773067.8, 2022334150, 3,229,948, 202280071911.6, 311000, 202447016521, 2024-510617, 10-2024-7009789, 20771386.8, 2020337921, 3151339, 202080073591.9, 202217016880, 2022-513074, 10-2022-7009541) and the associated authors are LCR, DFR, and ARD. The authors are also inventors on other patents and patent applications, which span the following topics: treatments for pulmonary arterial hypertension and vasculopathy (2879682, 2013296611, 2018201231, 10,071,123, 11,666,602, 2,880,808, 237021, 344280 , 6901823, 2143255, 2019-045535, 2879684, 3492084, 10,016,463, 10,842,823, 11,839,631, 12,310,992, 2880811, 3492084, 6746313, 6810717, 2143961, 3878452, 2943069, 3415150, 2014205557, 2017258866, 2019200504, 11,839,596, 10,080,730, 11,141,393, 12,274,684, 2897805, ZL201480008413.2, 239603, 255547, 347751, 6431850, 6709834, 10-2466885, 10-2348611), organ decellularization (12,022,823, 10,757,932, 3188595, 2959526, 385492, 6896615, 7110449, 10-2400425, 3,217,634), lung tissue engineering (11,648,280, 2393914, 10,188,683, 3,084,176, 2,751,133, ZL201080015120.9, 5824/DELNP/2011, 487876, 2019-156705, 6609595, 633777, 6042614, 10-1822662), tissue evaluation tools (17/943,728, 22797535.6, 2022345001, PCT/US2022/043302, 3,231,826, 202280075804.0, 311466, 202447028377, 2024-516519, 10-2024-7011899), mitochondrial treatment of organs for transplantation (16/903,975, 20739497.4, PCT/US2020/038133, 3,144,252, 202080058469.4, 202247002864, 2021-575381, 2025-140799, 10-2022-7000848), MSC immunomodulation (11,571,444, 3534917, 266175, 4442998, 7179723, 602017061040.2, 2017350732, 18/147,929, 3041514, 201780074352.3, 10-2019-7014254), extracellular vesicles (10,946,047, 2017283658, 473816, 17/109,775, 17734909.9, 201780047654.1, 263727, 2018-565684, 10-2019-7001666), endothelial glycocalyx repair (18/917,505, 2024364879, 63/590,949, PCT/US2024/051508, 3,308,482, 327852), and endothelial barrier (18/077,038, 22847023.3, 2022407019, PCT/US2022/052120, 3,240,212, 202280091251.8, 313328, 202447048237, 2024-534276, 10-2024-7022482). THP, RWB, SSH, and BAF are inventors on the aforementioned patents and patent applications. LCR, SSH, DFR, and RJO are preparing a patent application on 3-dimensional cell culture methods.

Supplemental Methods

Donor Information

Donor 9VX. Age: 35. Sex assigned at birth: male. Race: White. Smoking history: yes, unspecified pack-years.

Donor R0G. Age: 31. Sex assigned at birth: male. Race: Black or African American. Smoking history: none.

Donor D7V. Age: 44. Sex assigned at birth: male. Race: White. Smoking history: none.

Donor C70. Age: 44. Sex assigned at birth: male. Race: White. Smoking history: none.

Donor XH4. Age: 13. Sex assigned at birth: female. Race: White. Smoking history: none.

Donor N1H. Age: 46. Sex assigned at birth: male. Race: White. Smoking history: none.

Donor WB0. Age: 36. Sex assigned at birth: male. Race: White. Smoking history: none.

Donor JXB. Age: 30. Sex assigned at birth: female. Race: White. Smoking history: none.

Donor VRL. Age: 33. Sex assigned at birth: male. Race: White. Smoking history: yes, unspecified pack-years.

Donor K5F. Age: 32. Sex assigned at birth: male. Race: White. Smoking history: yes, 3 packs/week, unspecified pack-years.

Donor Y5R. Age: 58. Sex assigned at birth: male. Race: White. Smoking history: none.

Donor QL2: Age: 61. Sex assigned at birth: male. Race: White. Smoking history: none.

Donor G2X: Age: 29. Sex assigned at birth: male. Race: White. Smoking history: yes, 13 pack-years.

Donor PN6: Age: 41. Sex assigned at birth: female. Race: White. Smoking history: none.

Donor M3L: Age: 58. Sex assigned at birth: female. Race: White. Smoking history: yes, unspecified pack-years.

Donor A8D: Age: 31. Sex assigned at birth: male. Race: White. Smoking history: none.

Donor W2V: Age: 39. Sex assigned at birth: male. Race: White. Smoking history: yes, 1 pack-year.

Donor SG3: Age: 61. Sex assigned at birth: male. Race: White. Smoking history: none.

Donor TN3: Age: 27. Sex assigned at birth: male. Race: White. Smoking history: yes, 1.5 packs/day, unspecified pack-years.

Donor V5M: Age: 34. Sex assigned at birth: male. Race: White. Smoking history: Yes, 8 pack-year.

Donor B9D: Age: 41. Sex assigned at birth: male. Race: White. Smoking history: none.

Donor P2J: Age: 55. Sex assigned at birth: female. Race: White. Smoking history: none.

Cell Culture

Organoid Culture

Matrigel was thawed at 4ºC and kept on ice while culture plates were placed in a 37 ºC, humidified incubator to warm. Cryopreserved AT2s were thawed and resuspended in T2-Max at a concentration of 500,000 cells/mL. The cell suspension was mixed with an equal volume of Matrigel and dispensed in 25 uL droplets to pre-warmed 48-well plates using an adaptation of the direct droplet method (2). The plate was returned to the incubator for at least 15 minutes to polymerize before adding 500 uL warmed T2-Max per well. Media was replaced every 2-3 days for a total of 12 days.

Short 2D AT2 Culture for Immunofluorescence Analysis

Glass-bottom plates were pre-coated using collagen-I diluted in DPBS for at least 1 hour at room temperature (RT) and rinsed before seeding cells. Cryopreserved isoAT2s or passage 2 AT2s were thawed and cryomedia was slowly diluted with T2-Max. Passage 2 AT2s were seeded in T2-Max at 2 x 10^4 - 1 x 10^5 cells/cm^2 and isoAT2s were seeded at 1-5 x 10^5 cells/cm^2. Cultures were incubated at 37 ºC for 4 to 20 hours before being processed for immunofluorescence microscopy, described below. For negative controls and non-alveolar identity markers, cryopreserved passage 2 primary, small airway epithelial cells (SAEP) and an enterocyte cell line, HT-29, were seeded directly into coated 96-well, glass-bottom plates before being processed for immunofluorescence microscopy.

Media, Stiffness, and Substrate Study Culture

Softwell, glass-bottom, or standard polystyrene culture plates were coated with 0.1% gelatin solution or 100 ug/mL PureCol diluted in DPBS for at least 1 hour at RT. Cryopreserved isoAT2s were thawed in T2-Max with 100 U/mL DNase I. An equivalent volume of DPBS was used to wash wells before seeding AT2s at a density of 5 x 10^4 HT2-280+ cells/cm^2 in T2-Max. To allow equivalent attachment between conditions, cultures were left undisturbed from seed (day 0) until day 2 at which time the medium was exchanged for either fresh T2-Max or T1-Base depending on the experimental condition. EdU was spiked into the medium at a final concentration of 3 μM on day 3 for 24-well plate cultures and allowed to incubate for 24 hours. Cultures were harvested on day 4 either by direct lysis in Buffer RLT for RNA isolation (6-well plates) or by paraformaldehyde fixation for EdU, HT2-280, and pSP-C staining (24-well plates). IsoAT2 cells from six donors were evaluated across two independent experiments.

2D Multi-passage AT2 Culture

175 cm^2 cell culture flasks were coated with 20 ug/mL rat tail collagen-I and cryopreserved isoAT2 cells from 4 donors were thawed in T2-Max. After washing culture flasks, isoAT2 were seeded at a density of 30,000 cells/cm^2 in 50 mL T2-Max with media exchanges every 2-3 days. Confluent cells were harvested via brief trypsin exposure before the cell culture flask was incubated at 37 ºC for 5-10 minutes. Cells were

collected in chilled DPBS + 5% FBS, pelleted at 300 x g for 5 minutes, and resuspended in 5 mL of T2-Max. Cell count and viability were analyzed using a Nexcelom Cellometer. Following harvest, AT2 cells were seeded into a new T-175 flask at 10,000 cells/cm^2 and this process was repeated through passage 2 for all donors.

<u>SEM Cultures</u>

To generate samples for SEM analysis of cell thickness, collagen-I coated transwells were seeded with passage 2 AT2s as described above. Cells were cultured in T2-max for 2 days, followed by T1-Diff for 3 days, then exchanged for T1-Base. After 2 days in T1-Base, only medium in the transwell insert was exchanged with either fresh T1-Base medium (AT1 only inserts) or a hybrid medium containing 20,000 freshly thawed AT2 cells (AT1 & AT2 inserts). The hybrid medium was composed of 60% T1-Base and 40% T2-Max. Medium in the wells of the 24-well plate was not exchanged and remained as T1-Base in both conditions. The next day, inserts were washed, fixed with 3.7% buffered paraformaldehyde for 15 minutes, and stored at 4°C in DPBS prior to sample preparation.

Immunofluorescence-based Assays

<u>Flow Cytometry</u>

For all cultured cells, phenotype analysis was performed post-cryopreservation. Cryovials were thawed, T2-Max was added, and 1 x 10^6 cells were transferred to a conical tube. Cells were then washed with DPBS and pelleted by centrifugation at 300 x g. Cells were resuspended in DPBS before adding an equivalent volume of 4% paraformaldehyde, fixed for 15 minutes at RT, and washed in DPBS. Cell staining was performed by incubating 5 x 10^5 cells overnight at 4 °C with one of the following antibody combinations. For data in figure S2D, cells were stained in FACS buffer (1% BSA, 1 mM EDTA) with anti-EPCAM-FITC or mouse IgG1-FITC isotype control antibody. Cells were washed with FACS buffer and resuspended in FACS buffer for analysis on a BD FACSLyric instrument. For data in figure S4B, the antibody combinations for staining were as follows: anti-HT2-280 and anti-EPCAM-FITC in DPBS, anti-

pSP-C in DPBS+0.1% Triton X-100, anti-CK5 in DPBS+0.1% Triton X-100, anti-mouse isotype control and anti-mouse IgG-FITC, or anti-rabbit isotype control in DPBS+0.1% Triton X-100. Following incubation with primary antibodies, cells were washed with DPBS and incubated with respective conjugated secondary antibodies, if applicable, for 30 minutes at RT. Cells were again washed and resuspended in DPBS for analysis. Flow cytometry was run on a BD FACSVerse instrument. FCS Express 7 was used for all flow data analysis. Gating of populations was based on isotype controls and location of gates were checked against the negative stained population.

EdU Assessment of IsoAT2 Banks

Cryopreserved cells were thawed and slowly diluted with T2-Max. After counting and viability assessment, cells were seeded into BioCoat 24-well plates at 5 x 10^4 cells/cm^2, and cells were allowed to attach for 3 hours in an incubator before adding an equivalent volume of 20 µM EdU. After 48 hours in culture, cells were fixed and processed according to the vendor's Click-iT EdU Imaging Kits Protocol. Plates were imaged on a Revvity Celigo Image Cytometer and analyzed using the Expression Analysis application. Nuclei were used as the mask to define total cells. The histogram function was used to separate populations based on EdU mean intensity with a threshold set at the trough between positive and negative populations. The fraction of cells greater than this threshold was reported as percent EdU positive.

EdU Assessment of Microcarrier Cultures

Microcarrier (MC) culture samples were aseptically taken from a bioreactor and 15 mL of cell suspension was transferred to a 50 mL conical tube containing a vented cap. Pre-warmed T2-Max containing 50 µM EdU was added to achieve a 10 µM EdU final concentration. Cell-laden MCs were incubated with EdU for 6 hours in an incubator while shaking. Following EdU incubation, cell-laden MCs were washed twice with DPBS and then fixed with paraformaldehyde. Fixation, processing, and imaging are described in the Immunofluorescence Microscopy section.

Assessment of Media, Stiffness, and Substrate Cultures

For EdU detection in the study of factors affecting the expansion and stability of cultured AT2s, freshly diluted 4% paraformaldehyde (ThermoFisher) solution was added to wells at the end of culture and incubated for 15 minutes at RT. Blocking and permeabilization were done using 0.5% BSA in DPBS with 0.25% Triton-X for 15 minutes at RT. EdU labeling was performed using the vendor's Click-iT EdU Imaging Kit Protocol, followed by immunofluorescent staining with mouse HT2-280 (1:150) and rabbit pro-SP-C (1:1000) diluted in DPBS with 1% BSA. After a 1 hour incubation with primary antibodies, plates were washed in BSA buffer three times before applying secondary antibodies diluted 1:1000 in DPBS with 1% BSA for 1 hour. Plates were washed in BSA buffer three times before incubation with Hoechst diluted 1:5000 in DPBS. Images were acquired on a Revvity Celigo Image Cytometer and analyzed using the Expression Analysis application. Nuclei were used as the mask to define total cells, and the scatter plot function was used to separate populations by EdU and HT2-280 expression. Thresholds for each channel were set at two times the background value in EdU- and HT2-280-negative cells. The percentage of cells in each quadrant was extracted and normalized by ratio to the control condition, gel-T2-soft.

Immunofluorescence Microscopy

Samples, inclusive of multi-well plates, chamber slides, and cell-laden MCs, were washed in DPBS and then fixed for 15 minutes at RT in 2% paraformaldehyde in DPBS. Samples were permeabilized with 0.1% Triton X-100 in DPBS containing 2% BSA and incubated in blocking solution for greater than 2 hours (DPBS+10% Normal Goat Serum+2% BSA+0.01% Triton X-100). Samples were incubated overnight with primary antibodies in BSA Buffer (DPBS+2% BSA+0.01% Triton X-100) at 4 ^{o}C, while mixing. Samples were incubated for 1 hour with secondary antibodies, with or without Phalloidin, in BSA Buffer at RT while mixing. For samples requiring EdU conjugation, the Click-iT reaction was performed, per the manufacturer's protocol, prior to primary antibody incubation. Following each antibody incubation step, samples were washed with BSA Buffer 3 times for 5 minutes while mixing.

For multi-well plates and chamber slides, wash buffer was exchanged for DPBS containing nuclear counterstain and samples were imaged on a Leica Stellaris5 confocal, a Revvity Celigo Image Cytometer, a Revvity Opera Phenix Plus or a Leica DMI6000 SD inverted widefield microscope. For AT1 size quantification and marker expression analysis, Ibidi multi-well plates were imaged with the Opera Phenix Plus, confocal z-stacks were turned into maximum intensity projections and stitched into global images in Harmony. Using Harmony image analysis tools, nuclei and cell boundaries were first identified and then area of each cell was quantified along with fluorescence intensity of each cell or nucleus. The mean fluorescence intensity (MFI) and intensity sum (sum fluorescence intensity or SFI) were exported for each cell, and the data from 2-3 wells was averaged for each donor. To determine RAGE, nuclear-associated active YAP, HT1-56, GPRC5a, and caveolin-1 positivity, donor MFI was calculated for each target, the day 1 MFI of the 3 donors was then averaged, and the threshold for positive expression was set as the day 1 average MFI + 1 standard deviation. A similar Harmony workflow was used for AT2 pSP-C and SP-D quantification through nuclei and cell boundary detection, after which secondary only controls were used to define intensity thresholds for HT2-280, pSP-C and SP-D positivity. For pSP-C, in addition to secondary only controls, intensity thresholds were set above SAEP negative control cells and dim positive cells in the AT2 cultures. The select population analysis building block was utilized to define cells based on those intensity thresholds as positive for both, none, or either protein in each staining combination (HT2-280 and pSP-C or HT2-280 and SP-D).

For cell-laden MC samples, after the final wash, MCs were resuspended in mounting medium (equal volumes 80% Glycerol/20% DPBS:Prolong Gold DAPI Mountant), transferred to a black-walled 96-well imaging plate, and imaged using a Leica Stellaris5 confocal microscope.

Image processing was conducted in Zeiss Zen, Leica LASX or Revvity Harmony software. Brightness was adjusted for ease of visualization while maintaining dynamic range and contrast was adjusted to minimize background signal. The brightness and contrast settings were held constant for each staining combination in an image panel. Gamma adjustments were not applied to any image.

Electron Microscopy

For scanning electron microscopy, transwell membranes were cut from supports and cells were dehydrated through an ethanol gradient (2 x 30-minute successive washes in 50%, 75%, and 100% ethanol) and dried using automatic settings in a critical point dryer. Samples were then coated with 10-15 nm gold using a MINIQS sputter coater and imaged on a ThermoFisher PhenomProX desktop SEM.

For transmission electron microscopy, cells were cultured on microcarriers as previously described. On day 5, media was exchanged with fresh media and cells were cultured for an additional 72 hours. Cell-laden microcarriers were then separated from conditioned media and fixed. Surfactant in the conditioned media was then pelleted at 1000 x g for 10 minutes and the media pellet was fixed. Fixed microcarriers and media pellets were stored in fixative at 4 °C until processing. TEM samples were processed and imaged at a core facility at the University of North Carolina. The TEM fixative used was 2% paraformaldehyde 2.5% glutaraldehyde in 0.1M sodium cacodylate buffer at pH 7.4

Karyotyping

G-banded karyotyping, which is a cytogenetic technique used to assess chromosomal number and structure at the single-cell level, was performed by Wicell Research Institute. Briefly, actively dividing cells were cultured and arrested in metaphase when chromosomes were condensed. Cells were then detached from culture vessels, treated with hypotonic solution, and then fixed using Carnoy's fixative. After fixation, the cells were dropped onto microscope slides, treated with trypsin, and banded with Leishman's stain, producing a characteristic pattern of light and dark bands unique to each chromosome. These banding patterns, analyzed under a microscope by trained cytogenetic technologists, allowed for the detection of structural abnormalities (such as translocations, deletions, duplications, and inversions) as well as numerical changes (aneuploidy, polyploidy). Results were reported according to the International System for Human Cytogenomic Nomenclature.

Western Blot Analysis

Cryopreserved passage 2 AT2s from 3 donors were thawed in T2-Max and incubated on ice with RIPA lysis buffer and protease inhibitor cocktail. Cell lysates were cleared by centrifugation at 14,000 x g for 10 minutes at 4 ºC and supernatants were stored at -80 ºC until analysis. Protein bands were resolved on Novex™ 10 to 20%, Tricine, 1.0 mm, Mini Protein Gels at 125V in Tricine SDS Running Buffer and transferred to a PVDF membrane using the iBlot 3 Western Blot Transfer System. Membranes were blocked in PBST Buffer (DPBS with 0.05% Tween-20) + 5% non-fat milk for 1 hour at RT followed by overnight incubation at 4 ºC in PBST + 1% non-fat milk and primary antibodies against mSP-C (1:5000), pSP-C (1:1000), or GAPDH (1:1000). Membranes were washed with PBST and incubated in PBST + 1% non-fat milk and goat anti-rabbit or goat anti-mouse IgG-HRP (1:2000 for pSP-C and GAPDH, 1:5000 for mSP-C) for 1 hour at RT. Membranes were washed in PBST and visualized using Pierce™ ECL Western Blotting Substrate (pSP-C and GAPDH) or SuperSignal™ West Atto Ultimate Sensitivity Substrate (mSP-C) on the Syngene G:BOX Chemiluminescent Imager.

Surfactant HPLC

AT2 cells were cultured on microcarriers, as described herein. On day 5, media was exchanged with fresh media and cells were cultured for an additional 72 hours. Conditioned media samples were collected from the bioreactor immediately after media exchange on day 5 and at 48- and 72-hours post-media exchange, days 7 and 8, respectively. Microcarriers were allowed to settle, and the supernatant was transferred to new tubes and stored at -80 ºC prior to analysis.

Circulating SP-B was evaluated using LC-MS/MS analysis at Alera Labs. Briefly, protein denaturation was done using trypsin digestion at 60°C. Peptides were then analyzed via LC-MS/MS. SPB peptide was spiked in and sample concentration calculated based on the resulting standard curve.

Gene Expression Analysis

Total mRNA was isolated using RNeasy Mini kits following the manufacturer's protocol and each sample was eluted in 35-50 uL TE buffer. For cDNA synthesis reactions, a total of 500-1000 ng of input RNA

was used following manufacturer's protocol. The resulting complementary DNA was diluted with nuclease-free water to a working concentration of 2.5-5 ng/uL for use in real-time PCR reactions. Taqman Fast Advanced Master Mix and a ThermoFisher QuantStudio 6 thermal cycler were used to perform real-time PCR in 96-well plates with "Fast" cycling parameters. Predesigned TaqMan gene expression assays were used for all reactions. A total of 40 cycles were performed on replicate wells for each sample, and relative fold changes to experimental controls were calculated using the delta-delta Ct method.

For mRNA profiling on a NanoString nCounter Pro system, a total of 150 ng input RNA was used for hybridization with a custom codeset that included probes specific to AT2/transitional/AT1 genes and 8 housekeeping genes for normalization. The nCounter Pro Analysis System automated workflow was used for post-hybridization processing and digital data acquisition. Raw counts were imported to Rosalind for data QC and normalization with an average image quality score of 0.97 from 555 FOVs per sample, an average binding density of 1.18, and an average positive control linearity of 0.99 across 24 samples. Log2 normalized counts were used to generate relevant heat maps.

scRNA-seq Library Preparation and Next-generation Sequencing

The AT2 expansion samples were processed at SingulOmics using the 10x Genomics Chromium platform 3' v4 chemistry resulting in ~6,000 cells per sample. The recell FFPE tissues were dissociated into single-cell suspensions by SingulOmics using the gentleMACS Octo Dissociator and the 10x Genomics Chromium X system. The Chromium Single Cell Gene Expression Flex kit for multiplex samples was then used to construct single cell fixed RNA libraries. The process resulted in ~9,600 cells per sample. Next-generation sequencing of both datasets was performed on an Illumina NovaSeq X Plus sequencer, and reads were aligned to the GRCh38 transcriptome using the CellRanger v9.0 pipeline with default parameters. Introns were included in the analysis. Cells were removed if they contained fewer than 500 RNA molecules, fewer than 700 or greater than 10,000 expressed genes, and greater than 10% of reads arising from mitochondrial genes.

scRNA-seq Sample Integration and Cell Type Annotation

Seurat v5 (3) was used to analyze all filtered CellRanger output counts. For each dataset, individual samples were normalized using the sctransform v2 pipeline, and dimensionality reduction of the merged samples was performed with PCA. Samples were then integrated with Harmony integration (4) as implemented within the Seurat package. Cells were visualized with UMAP based on the Harmony embeddings. Both datasets were subjected to Louvain clustering using the Seurat FindNeighbors function with 20 dimensions and the FindClusters function with a resolution of 0.2. Gene markers were obtained for each cluster using the Wilcoxon Rank Sum Test implemented with Seurat FindAllMarkers() function. Cluster-based cell types were annotated for the AT2 expansion dataset by comparing cluster markers with canonical cell type markers. Cell type labels were also predicted for both datasets with Seurat's anchor-based label transfer method using a reference scRNA-seq dataset. A reference was chosen that had good representation of alveolar cells from human lung tissue, including an annotation of Transitional AT2 cells (1). The full reference dataset was used and contained 114,396 cells among 31 different cell types. Finally, for any direct comparison between the AT2 expansion and recell datasets, samples from both datasets were merged and integrated with Harmony integration.

scRNA-seq Differential Gene Expression Analysis

Differential gene expression analysis was performed between the isoAT2 and P2 conditions in the AT2 expansion scRNA-seq dataset. To account for within-sample correlations between cells, each sample was aggregated into a pseudobulk sample (n = 3 for each condition) using Seurat's AggregateExpression function. Resulting sample counts were subjected to the edgeR pipeline (5) for differential expression identification using their GLM quasi-likelihood F-tests. Differentially expressed genes (DEGs) were defined as those with an FDR p-value less than 0.05 and a log2 fold change greater than 1.0. For pathway enrichment analysis, DEGs up-regulated in each condition were further restricted to those expressed by at least 10% of the cells. These final DEGs were tested for pathway over-representation using the Panther Classification System (6) Overrepresentation Test (Released 20240807) with the *Homo sapiens* reference

list against the Reactome database (7) version 86 (Released 2023-09-07). The Fisher's Exact test was used, and false discovery rate was calculated. Significantly over-represented pathways were identified with FDR < 0.05 and ranked by fold enrichment.

VEGF-A ELISA

Conditioned media was collected from expanded AT2 cultures taken through the AT1 differentiation protocol. VEGF-A ELISAs were performed according to the manufacturer's instructions. For the donor comparison data set, secreted VEGF-A concentration was normalized to nuclei counts taken from a 24-well plate cultured in tandem.

Recellularized Tissue Assessment

The engineered lungs were fixed with 10% neutral-buffered formalin instilled via the airway under a gravity pressure head of 25 cmH_2O for 4 hours. The tissue specimens were dissected and embedded in paraffin, sectioned at 5 µm, stained with hematoxylin and eosin (H&E), and imaged with a Zeiss Axioscan 7 slide scanner. Immunofluorescence staining was performed after standard deparaffinization and antigen retrieval in pH 6.0 citrate buffer. Slides were blocked for 15 minutes in Background Buster before incubating overnight at 4°C with primary antibodies to HT2-280 (1:150), pro-SP-C (1:1000), GPRC5A (1:500), HT1-56 (1:200), Caveolin-1 (1:100), EMP2 (1:200) or RAGE (1:100) diluted in 1% BSA solution. Slides were washed three times in TBS before incubating with isotype-specific secondary antibodies diluted 1:1000 in 1% BSA solution for 1 hour at RT. After three washes in TBS, slides were mounted with Prolong Gold Antifade plus DAPI and scanned with a Zeiss Axioscan 7 slide scanner or imaged on a Leica Stellaris5 confocal microscope. Image processing was conducted in Zeiss ZEN or Leica LASX software. Brightness was adjusted for ease of visualization while maintaining dynamic range, and contrast was adjusted to minimize background signal. The brightness and contrast settings were held constant for each staining combination in an image panel. Gamma adjustments were not applied to any image.

Recell Immunohistochemistry Quantification

Epithelial coverage analysis and immunostaining quantification was performed in QuPath v0.5.1. For epithelial coverage analysis, tissue was detected in a semi-automated manner using a pixel classifier and pleural extracellular matrix (ECM) or small tissue regions at the edge of the slide exhibiting dim edge effect-related staining were excluded from analysis. Scaffold area was detected using a pixel classifier to measure scaffold area based on collagen IV staining. This annotation was further classified as either covered by Ck8-positive epithelial cells, or uncovered ECM. Area of Ck8 staining was divided by total area of collagen IV ECM to generate percent of the total scaffold covered by epithelial cells. For immunostaining quantification, cell detection was first performed to identify each DAPI-labelled nuclei with a 4 um expansion around the nucleus as an object. For Ki67-stained tissue sections, object detection with a single threshold for mean nuclear intensity was used with a value determined in secondary only controls. For pSP-C and RAGE quantification, semi-automated object classification was performed by selecting positive and negative cells for each marker and then further refined across samples stained with primary and secondary antibodies or secondary only controls to ensure signal specificity. Counts for positive objects (cells) were divided by the total number of objects (cells) detected and reported as a percentage.

Recell Tissue RNAscope

Analysis of Human Advanced Glycosylation End Product-Specific Receptor (*AGER*) and Surfactant Protein C (*SFTPC*) mRNA using RNAscope LS Multiplex Fluorescent ISH/IF Assay was done using the RNAscope^{TM} LS Multiplex Fluorescent Reagent Kit at Advanced Cell Diagnostics (ACD, a Bio-techne brand). Briefly, 5 µm formalin-fixed, paraffin-embedded tissue sections were pretreated with heat and protease prior to hybridization with the target oligo probes. Preamplifier and amplifier were hybridized sequentially, followed by a TSA-fluorophore reaction. Specific RNA staining signal was identified as fluorescent, punctate dots, which correlate to the number of individual RNA molecules, whereas dot intensity reflects the number of probe pairs bound to each molecule. Percentage of positive cells was

scored visually by a qualified scientist at ACD and was based on number of cells with >1 dot/cell and results were binned into categories (i.e., 0%, 1-25%, 26-50%, 51-75%, 76-99%, 100%). Each sample was also assigned a score according to the following criteria: 0 (no staining or <1 dot/10 cells), 1 (1-3 dots/cell), 2 (4-9 dots/cell, no or very few dot clusters), 3 (10-15 dots/cell and/or <10% dots are in clusters), or 4 (>15 dots/cell and/or >10% dots are in clusters).

Reagent Information

REAGENT	SOURCE	IDENTIFIER
**Antibodies**		
Rabbit polyclonal anti-Mature SP-C	Seven Hills	Cat# WRAB-76694; RRID: AB_2938817
Rabbit polyclonal anti-SP-C	Sigma-Aldrich	Cat# HPA010928; RRID: AB_1857425
Mouse monoclonal anti-HT2-280	Terrace Biotech	Cat# TB-27AHT2-280; RRID: AB_2832931
Mouse monoclonal anti-HT1-56	Terrace Biotech	Cat# TB-29AHT1-56; RRID:AB_2847898
Rabbit anti-Ki-67	Abcam	Cat# ab16667; RRID:AB_302459
Mouse anti-EpCAM FITC	Agilent/Dako	Cat# F086001; RRID:AB_578689
Mouse anti-p40 (ΔNp63) [Clone BC28]	Biocare Medical	Cat# ACI 3066 A; RRID: AB_2858274
Rabbit anti-Cytokeratin 5	Abcam	Cat# ab52635; RRID:AB_869890
Rabbit monoclonal anti-ZO-1	Cell Signaling	Cat# 13663; RRID: AB_2798287
Rabbit monoclonal anti-Claudin 18	Invitrogen/ThermoFisher	Cat# 700178; RRID: RRID: AB_2532290
Rabbit monoclonal anti-active YAP	Abcam	Cat# ab205270; RRID: AB_2813833
Rabbit polyclonal anti-GPRC5A	Sigma-Aldrich	Cat# HPA007928; RRID: AB_1849318
Mouse monoclonal anti-Caveolin-1	Invitrogen/ThermoFisher	Cat# MA3-600; RRID: AB_779568
Rabbit monoclonal anti-RAGE	Abcam	Cat# ab216329; RRID: AB_2884897
Rabbit polyclonal anti-EMP2	Novus Biologicals	Cat# NBP1-86847; RRID: AB_11030439
Rabbit monoclonal anti-NKX2-1	Abcam	Cat# ab227652; RRID: AB_3096124
Mouse monoclonal anti-SOX9	Invitrogen/ThermoFisher	Cat# H00006662-M02; RRID: AB_877610

Mouse monoclonal anti-LAMP3	Novus Biologicals	Cat# DDX0190P;<br>RRID: AB_3107396
Mouse monoclonal anti-SP-D	R&D Systems	Cat# MAB1920;<br>RRID: AB_2185521
Mouse monoclonal anti-Cytokeratin 14	Abcam	Cat# ab7800;<br>RRID: AB_306091
Mouse monoclonal anti-Cytokeratin 17	Invitrogen/ThermoFisher	Cat# MA1-06325;<br>RRID: AB_559766
Rat monoclonal anti-Cytokeratin 17	Biolegend	Cat# 697202;<br>RRID: AB_2687136
Guinea Pig anti-Cytokeratin 8	LS Bio	Cat# LS-C193787;<br>RRID: NA
Mouse monoclonal anti-SOX2	Invitrogen/ThermoFisher	Cat# MA1-014;<br>RRID: AB_2536667
Mouse monoclonal anti-P53	Invitrogen/ThermoFisher	Cat# MA5-12571;<br>RRID: AB_10986581
Rat monoclonal anti-Galectin 3	Invitrogen/ThermoFisher	Cat# 14-5301-82;<br>RRID: AB_837132
Rabbit polyclonal anti-Claudin 4	Invitrogen/ThermoFisher	Cat# 36-4800;<br>RRID: AB_253326
Mouse monoclonal anti-GAPDH	Cell Signaling	Cat# 97166<br>RRID: AB_2756824
Mouse IgM Isotype Control	Abcam or<br>BD Biosciences	Cat# ab91545 or<br>Cat# 557275;<br>RRID:AB_479595
Mouse IgG1 FITC Isotype Control	Agilent/Dako	Cat# X092701-5
Mouse IgG1k - Isotype Control	Abcam	Cat# ab170190;<br>RRID:AB_2736870
Rabbit IgG – Isotype Control	Abcam	Cat# ab171870 ;<br>RRID:AB_2687657
Goat anti-Rabbit IgG (H+L) Alexa Fluor 488	Invitrogen/ThermoFisher	Cat# A-11034;<br>RRID:AB_2576217
Goat Anti-Mouse IgM (μ) Alexa Fluor 647	Abcam	Cat# ab150123;<br>RRID:AB_2893175
Goat anti-Mouse IgG1 Alexa Fluor 647	Invitrogen/ThermoFisher	Cat# A-21240;<br>RRID:AB_2535809
Goat anti-Mouse IgG (H+L) Alexa Fluor 555	Invitrogen/ThermoFisher	Cat# A21422;<br>RRID:AB_2535844
Goat anti-Rabbit IgG (H+L) Alexa Fluor 555	Invitrogen/ThermoFisher	Cat# A21428;<br>RRID:AB_2535849
Goat anti-Mouse IgM Alexa Fluor 488	Invitrogen/ThermoFisher	Cat# A-21042<br>RRID:AB_2535711
Goat anti-Rat IgG2a FITC	Invitrogen/ThermoFisher	Cat# PA1-84761<br>RRID:AB_933936
Goat anti-Mouse IgG2b Alexa Fluor 647	Invitrogen/ThermoFisher	Cat# A-21242<br>RRID:AB_2535811
Goat anti-Rabbit IgG (H+L) HRP	Abcam	Cat# ab205718;<br>RRID: AB_2819160
Goat anti-Mouse IgG (H+L) HRP	Abcam	Cat# ab205719;<br>RRID: AB_2755049
CD45 Microbeads, human	Miltenyi Biotec	Cat# 130-045-801
CD90 Microbeads, human	Miltenyi Biotec	Cat# 130-096-253
CD271 Microbeads, human	Miltenyi Biotec	Cat# 130-099-023
**Chemicals, peptides, and recombinant proteins**		

ProLong Gold Antifade Mountant with DAPI	Invitrogen/ThermoFisher	Cat# P36941
Alexa Fluor Plus 555 Phalloidin	ThermoFisher	Cat# A30106
Lysis 1 Acidic Lysis Buffer	Chemometec	Cat# 910-0010
A83-01	MCE or Stemcell Technologies	Cat# HY-10432 or Cat# 72024
CHIR99021	MCE or Stemcell Technologies	Cat# HY-10182 or Cat# 72054
DMH1	MCE or Stemcell Technologies	Cat# HY-12273 or Cat# 73634
Y-27632	MCE or Stemcell Technologies	Cat# HY-10583 or Cat# 72308
rEGF (human)	MCE or Stemcell Technologies	Cat# HY-P7109 or Cat# 78006
rKGF/FGF-7 (human)	MCE or GeminiBio	Cat# HY-P70597 or Cat# 300-861P
Fetal Bovine Serum	Gibco/ThermoFisher	Cat# 26140079
Primocin	Invivogen	Cat# ANT-PM-2
Antibiotic-antimycotic	ThermoFisher	Cat# 15240096
Gentamicin sulfate	GeminiBio	Cat# 200-336-0080
Amphotericin B	GeminiBio	Cat# A1316-10Y
DMEM/F12 + GlutaMAX	Gibco/ThermoFisher	Cat# 10565018 or
TrypLE Enzyme, no phenol red	Gibco/ThermoFisher	Cat# 12604021 or
DPBS	Gibco/ThermoFisher	Cat# 14190342 or
CryoStor CS10	Stemcell Technologies	Cat# 07930
DNase I	Worthington Biochemical	Cat# LS002140
Elastase	Worthington Biochemical	Cat# LS002298
HBSS (1X) [-] $MgCl_2$ [-] $CaCl_2$	Gibco	Cat# 14175-079
HBSS (1X) [+] $MgCl_2$ [+] $CaCl_2$	Gibco	Cat# 14025-076
Collagenase type IV	Worthington Biochemical	Cat# LS004188
Matrigel – growth factor reduced	Corning	Cat# 356231
0.1% Gelatin in water	Stem Cell Technologies	Cat# 07903
PureCol Solution	Advanced Biomatrix	Cat# 5005-100ML
TE, pH 7.0, RNase-free	ThermoFisher	Cat# AM9861
TRULI (Lats-IN-1)	MedChem Express	Cat# HY-138489
Collagen I, Rat Tail	Corning	Cat# 354236
Trypsin-EDTA (0.25%), phenol red	Gibco	Cat# 25200072
Citrate Buffer, pH 6.0, 10x	Sigma	Cat# C9999
Background Buster	Innovex Biosciences	Cat# NB306
16% Formaldehyde Solution (w/v), Methanol-free	ThermoFisher	Cat# 28908
Paraformaldehyde 4% aqueous solution, EM grade	Electron Microscopy Sciences	Cat# 157-4-100
2% Para 2.5% Glut In 0.1M Sodium Cacodylate pH 7.4	Electron Microscopy Sciences	Cat# 15960-01
Bovine Serum Albumin (IgG-Free, Protease-Free)	Jackson ImmunoResearch	Cat# 001-000-162
EDTA, 0.5M, pH 8.0	ThermoFisher	Cat# 15575020
**Commercial assays**		
Click-iT EdU Cell Proliferation Kit for Imaging, Alexa Fluor 647 dye	Invitrogen / ThermoFisher	Cat# C10340
Qiagen RNeasy Mini kit	Qiagen	Cat# 74106

High-Capacity cDNA Reverse Transcription Kit	Applied Biosystems/ ThermoFisher	Cat# 4368814
TaqMan™ Fast Advanced Master Mix	Applied Biosystems/ ThermoFisher	Cat# 4444965
RIPA Lysis Buffer, 10X	Millipore Sigma	Cat# 20-188
Halt™ Protease Inhibitor Cocktail, EDTA-Free	ThermoFisher	Cat# 78425
Pierce™ BCA Protein Assay Kits	ThermoFisher	Cat# 23227
Novex™ 10 to 20%, Tricine, 1.0 mm, Mini Protein Gels	ThermoFisher	Cat# EC6625BOX
Novex™ Tricine SDS Running Buffer (10X)	ThermoFisher	Cat# LC1675
Novex™ Tricine SDS Sample Buffer (2X)	ThermoFisher	Cat# LC1676
NuPAGE™ Sample Reducing Agent (10X)	ThermoFisher	Cat# NP0004
Spectra™ Multicolor Low Range Protein Ladder	ThermoFisher	Cat# 26628
Tween™ 20 Surfact-Amps™ Detergent Solution	Thermofisher	Cat# 28320
Blot™ 3 Transfer Stacks, midi, PVDF	ThermoFisher	Cat# IB34001
Pierce™ ECL Western Blotting Substrate	ThermoFisher	Cat# 32106
SuperSignal™ West Atto Ultimate Sensitivity Substrate	ThermoFisher	Cat# A38556
Softwell 6-well plate	Matrigen	Cat# SW6-EC-12
Softwell 24-well glass plate	Matrigen	Cat# SW24G-EC-12
Softwell 96-well glass HTS	Matrigen	Cat# SW96G-HTS-EC-PK
µ-Plate 24 Well	Ibidi	Cat# 82426
µ-Plate 96 Well	Ibidi	Cat# 89627
Via2-Cassette	Chemometec	Cat# 941-0024
nCounter Panel	Nanostring	Custom codeset
175cm² U-Shaped Angled Neck Cell Culture Flask with Vent Cap	Corning	Cat# 431080
6-well Clear TC-treated Multiple Well Plates	Corning	Cat# 3506
6.5 mm Transwell with 0.4 µm Pore Polyester Membrane Insert	Corning	Cat# 3470
BioCoat™ Collagen I 24-well Clear Multiwell Plate	Corning	Cat# 354408
Human VEGF ELISA Kit	Abcam	Cat# ab100662
**Probes**		
TaqMan probe: CLIC5	ThermoFisher	Hs00213494_m1
TaqMan probe: ANKRD1	ThermoFisher	Hs00173317_m1
TaqMan probe: CLDN4	ThermoFisher	Hs00533616_s1
TaqMan probe: CLDN18	ThermoFisher	Hs00212584_m1
TaqMan probe: GPRC5A	ThermoFisher	Hs01551896_m1
TaqMan probe: PDPN	ThermoFisher	Hs00366766_m1
TaqMan probe: CAV1	ThermoFisher	Hs00971716_m1
TaqMan probe: AGER	ThermoFisher	Hs00542584_g1
TaqMan probe: LGALS3	ThermoFisher	Hs00173587_m1
TaqMan probe: PPIA	ThermoFisher	Hs04194521_s1
TaqMan probe: ABCA3	ThermoFisher	Hs00184543_m1
TaqMan probe: KRT17	ThermoFisher	Hs01588578_m1
TaqMan probe: NAPSA	ThermoFisher	Hs00362192_m1
TaqMan probe: SFTPA2	ThermoFisher	Hs00359837_m1
TaqMan probe: SFTPC	ThermoFisher	Hs00161628_m1
TaqMan probe: SFTPD	ThermoFisher	Hs01108490_m1

TaqMan probe: CLDN3	ThermoFisher	Hs00265816_s1
TaqMan probe: KRT8	ThermoFisher	Hs01670053_m1
TaqMan probe: KRT5	ThermoFisher	Hs00361185_m1
TaqMan probe: TP63	ThermoFisher	Hs00978340_m1
TaqMan probe: PDGFA	ThermoFisher	Hs00234994_m1
TaqMan probe: WNT7A	ThermoFisher	Hs01114990_m1
TaqMan probe: LAMA3	ThermoFisher	Hs00165042_m1
TaqMan probe: LAMB3	ThermoFisher	Hs00165078_m1
TaqMan probe: LAMC2	ThermoFisher	Hs01043717_m1
TaqMan probe: COL4A1	ThermoFisher	Hs00266237_m1
TaqMan probe: COL4A2	ThermoFisher	Hs05006309_m1
TaqMan probe: COL4A3	ThermoFisher	Hs01022502_m1
TaqMan probe: COL4A4	ThermoFisher	Hs01011868_m1
TaqMan probe: COL4A5	ThermoFisher	Hs01012435_m1
TaqMan probe: COL4A6	ThermoFisher	Hs00361494_m1
Homo sapiens advanced glycosylation end product-specific receptor (*AGER*) transcript variant 1 mRNA	N/A	NM-001136.4
Homo sapiens surfactant protein C (*SFTPC*) transcript variant 1 mRNA	N/A	NM_003018.3

Supplemental References

1. Habermann AC, et al. *Single-cell RNA sequencing reveals profibrotic roles of distinct epithelial and mesenchymal lineages in pulmonary fibrosis*. 2020.

2. Chiu MC, et al. Establishing Bipotential Human Lung Organoid Culture System and Differentiation to Generate Mature Alveolar and Airway Organoids. *Bio Protoc*. 2023;13(8):e4657.

3. Hao Y, et al. Dictionary learning for integrative, multimodal and scalable single-cell analysis. *Nat Biotechnol*. 2024;42(2):293–304.

4. Korsunsky I, et al. Fast, sensitive and accurate integration of single-cell data with Harmony. *Nat Methods*. 2019;16(12):1289–1296.

5. Robinson MD, McCarthy DJ, Smyth GK. edgeR: a Bioconductor package for differential expression analysis of digital gene expression data. *Bioinformatics*. 2010;26(1):139–140.

6. Mi H, et al. PANTHER version 14: more genomes, a new PANTHER GO-slim and improvements in enrichment analysis tools. *Nucleic Acids Res*. 2019;47(D1):D419–D426.

7. Jassal B, et al. The reactome pathway knowledgebase. *Nucleic Acids Res*. [published online ahead of print: November 6, 2019]. https://doi.org/10.1093/nar/gkz1031.
