# Supplementary figures and images for: Leveraging factors that control alveolar epithelial cell fate enables large-scale expansion for lung tissue engineering
Source: J Clin Invest. 2026 Jun 1;136(11):e188701. doi: 10.1172/JCI188701 (PMC13221229; doi:10.1172/JCI188701)

Full unedited blot for Figure 2F

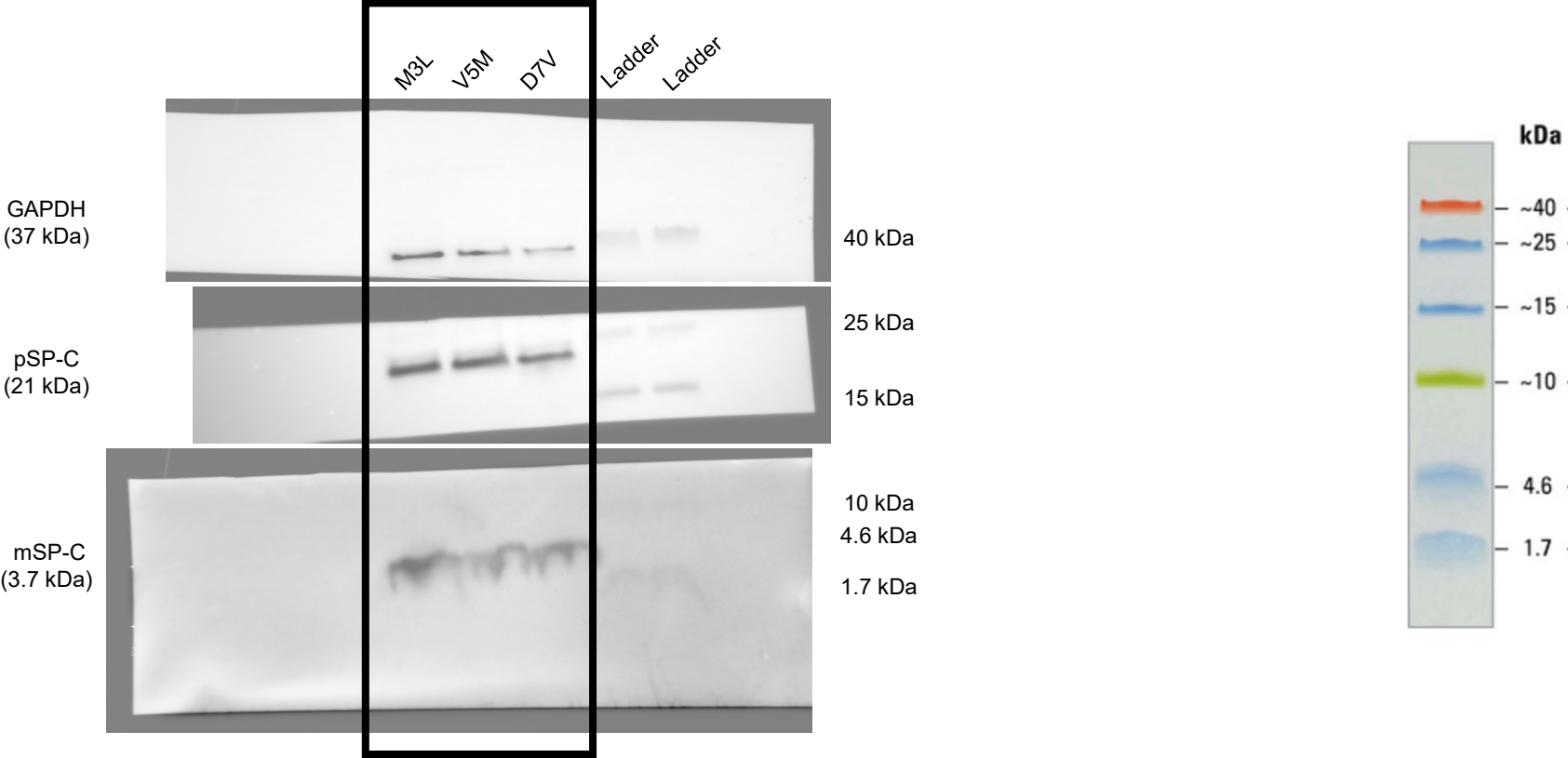

Supplement: Unedited blot and gel images [file jci-136-188701-s280.pdf]
